# Proceedings of the 12th annual deep brain stimulation think tank: cutting edge technology meets novel applications

**DOI:** 10.3389/fnhum.2025.1544994

**Published:** 2025-02-25

**Authors:** Alfonso Enrique Martinez-Nunez, Christopher J. Rozell, Simon Little, Huiling Tan, Stephen L. Schmidt, Warren M. Grill, Miroslav Pajic, Dennis A. Turner, Coralie de Hemptinne, Andre Machado, Nicholas D. Schiff, Abbey S. Holt-Becker, Robert S. Raike, Mahsa Malekmohammadi, Yagna J. Pathak, Lyndahl Himes, David Greene, Lothar Krinke, Mattia Arlotti, Lorenzo Rossi, Jacob Robinson, Bahne H. Bahners, Vladimir Litvak, Luka Milosevic, Saadi Ghatan, Frederic L. W. V. J. Schaper, Michael D. Fox, Nicholas M. Gregg, Cynthia Kubu, James J. Jordano, Nicola G. Cascella, YoungHoon Nho, Casey H. Halpern, Helen S. Mayberg, Ki Sueng Choi, Haneul Song, Jungho Cha, Sankaraleengam Alagapan, Nico U. F. Dosenbach, Evan M. Gordon, Jianxun Ren, Hesheng Liu, Lorraine V. Kalia, Sarah-Anna Hescham, Dorian M. Kusyk, Adolfo Ramirez-Zamora, Kelly D. Foote, Michael S. Okun, Joshua K. Wong

**Affiliations:** ^1^Norman Fixel Institute for Neurological Diseases, University of Florida, Gainesville, FL, United States; ^2^School of Electrical and Computer Engineering, Georgia Institute of Technology, Atlanta, GA, United States; ^3^Movement Disorders and Neuromodulation Centre, University of California San Francisco, San Francisco, CA, United States; ^4^Medical Research Council Brain Network Dynamics Unit, Nuffield Department of Clinical Neurosciences, University of Oxford, Oxford, United Kingdom; ^5^Departments of Biomedical Engineering, Electrical and Computer Engineering, Neurobiology and Neurosurgery, Duke University and Duke University Medical Center, Durham, NC, United States; ^6^Department of Neurobiology, Duke University Medical Center, Durham, NC, United States; ^7^Department of Neurosurgery, Duke University Medical Center, Durham, NC, United States; ^8^Center for Neurological Restoration, Cleveland Clinic, Cleveland, OH, United States; ^9^Department of Neurology, Cleveland Clinic Lerner College of Medicine of Case Western Reserve University, Cleveland, OH, United States; ^10^Weill Cornell Medical College, Feil Family Brain and Mind Research Institute, New York, NY, United States; ^11^Restorative Therapies Group Implantables, Research, and Core Technology, Medtronic Inc., Minneapolis, MN, United States; ^12^Department of Neurosurgery, University of California, Los Angeles, CA, United States; ^13^Boston Scientific Neuromodulation, Valencia, CA, United States; ^14^Neuromodulation Division, Abbott, Plano, TX, United States; ^15^NeuroPace, Inc., Mountain View, CA, United States; ^16^Newronika SpA, Milan, Italy; ^17^West Virginia University, Morgantown, WV, United States; ^18^Department of Bioengineering, Rice University, Houston, TX, United States; ^19^Department of Electrical and Computer Engineering, Rice University, Houston, TX, United States; ^20^Department of Neurology, Brigham & Women's Hospital, Harvard Medical School, Center for Brain Circuit Therapeutics, Boston, MA, United States; ^21^Institute of Clinical Neuroscience and Medical Psychology, Medical Faculty and University Hospital Düsseldorf, Heinrich Heine University Düsseldorf, Düsseldorf, Germany; ^22^Department of Neurology, Center for Movement Disorders and Neuromodulation, Medical Faculty and University Hospital Düsseldorf, Heinrich Heine University Düsseldorf, Düsseldorf, Germany; ^23^Wellcome Centre for Human Neuroimaging, UCL Queen Square Institute of Neurology, London, United Kingdom; ^24^Clinical and Computational Neuroscience, Krembil Research Institute, University Health Network, Toronto, ON, Canada; ^25^Faculty of Medicine, Institute for Neuromodulation and Neurotechnology, University Hospital Tübingen (UKT), University Tübingen, Tübingen, Germany; ^26^Department of Neurosurgery, Mount Sinai Medical Center, New York, NY, United States; ^27^Department of Neurosurgery, Maria Fareri Children's Hospital, Westchester Medical Center, Valhalla, NY, United States; ^28^Department of Neurology, Mayo Clinic, Rochester, MN, United States; ^29^Department of Neurology, Georgetown University Medical Center, Washington, DC, United States; ^30^Department of Biochemistry, Georgetown University Medical Center, Washington, DC, United States; ^31^Neuroethics Studies Program, Georgetown University Medical Center, Washington, DC, United States; ^32^Department of Psychiatry, Johns Hopkins University School of Medicine, Baltimore, MD, United States; ^33^Department of Neurosurgery, University of Pennsylvania, Philadelphia, PA, United States; ^34^Department of Surgery, Corporal Michael J. Crescenz Veterans Affairs Medical Center, Philadelphia, PA, United States; ^35^Nash Family Center for Advanced Circuit Therapeutics, Icahn School of Medicine at Mount Sinai, New York, NY, United States; ^36^Department of Radiology and Neurosurgery, Icahn School of Medicine at Mount Sinai, New York, NY, United States; ^37^Department of Neurology and Psychiatry, Icahn School of Medicine at Mount Sinai, New York, NY, United States; ^38^Department of Neurology, Washington University School of Medicine, St. Louis, MO, United States; ^39^Mallinckrodt Institute of Radiology, Washington University School of Medicine, St. Louis, MO, United States; ^40^Department of Psychological & Brain Sciences, Washington University, St. Louis, MO, United States; ^41^Department of Biomedical Engineering, Washington University, St. Louis, MO, United States; ^42^Program in Occupational Therapy, Washington University, St. Louis, MO, United States; ^43^Department of Pediatrics, Washington University School of Medicine, St. Louis, MO, United States; ^44^Department of Radiology, Washington University School of Medicine, St. Louis, MO, United States; ^45^Changping Laboratory, Beijing, China; ^46^Biomedical Pioneering Innovation Center, Peking University, Beijing, China; ^47^Edmond J Safra Program in Parkinson's Disease, Krembil Research Institute, Toronto Western Hospital, University Health Network, Toronto, ON, Canada; ^48^Division of Neurology, Department of Medicine, University of Toronto, Toronto, ON, Canada; ^49^School for Mental Health and Neuroscience, Maastricht University, Maastricht, Netherlands; ^50^Department of Neurosurgery, Maastricht University Medical Center, Maastricht, Netherlands; ^51^Department of Neurosurgery, RWTH Aachen University Hospital, Aachen, Germany

**Keywords:** neuromodulation, deep brain stimulation, Parkinson’s disease, epilepsy, sleep, stroke, depression, obsessive-compulsive disorder

## Abstract

The Deep Brain Stimulation (DBS) Think Tank XII was held on August 21st to 23rd. This year we showcased groundbreaking advancements in neuromodulation technology, focusing heavily on the novel uses of existing technology as well as next-generation technology. Our keynote speaker shared the vision of using neuro artificial intelligence to predict depression using brain electrophysiology. Innovative applications are currently being explored in stroke, disorders of consciousness, and sleep, while established treatments for movement disorders like Parkinson’s disease are being refined with adaptive stimulation. Neuromodulation is solidifying its role in treating psychiatric disorders such as depression and obsessive-compulsive disorder, particularly for patients with treatment-resistant symptoms. We estimate that 300,000 leads have been implanted to date for neurologic and neuropsychiatric indications. Magnetoencephalography has provided insights into the post-DBS physiological changes. The field is also critically examining the ethical implications of implants, considering the long-term impacts on clinicians, patients, and manufacturers.

## Introduction

The 12th Annual Deep Brain Stimulation (DBS) Think Tank was held from August 21st to 23rd, 2024 at the Fixel Institute Campus at the University of Florida Gainesville. The sessions were broadcast live and recorded for online access.

This Think Tank involved a broad number of topics and invited authorities from different backgrounds and expertise, including clinicians, scientists, engineers, members of industry and ethicists. To date, an estimate of 300,000 DBS devices have been implanted around the world.

The theme for the DBS Think Tank meeting was “Emerging indications and novel applications of DBS,” and it was divided into the following sections: movement disorders, stroke, traumatic brain injury, sleep, magnetoencephalography-DBS research, epilepsy, neuroethics in neuromodulation, mood and neuropsychiatric disorders, and emerging techniques. Additionally, the keynote speaker, Christopher Rozell from Georgia Tech presented on the use of artificial intelligence (AI) to analyze complex time series data as an emerging method to optimize neuromodulation.

### Looking for latent variables: lessons from NeuroAI to advance neuromodulation

Simultaneous advances in neurotechnology and data science have created new opportunities to advance neuromodulation using objective brain signal measurement. While promising in principle, the practical challenge of this approach has been heterogenous patient populations with vast arrays of different symptoms. Thus, instead of directly measuring the intended specific abnormality, we observe shadows of presumed underlying stereotyped circuit deficits that may appear differently across time or between individuals. Christopher Rozzell’s group proposes that we can learn from the long history of neuro-artificial intelligence (neuroAI), where research in (biological and computer) vision shares a similar challenge of attempting to infer the presence of interacting underlying causes drawn from potentially ambiguous datasets. NeuroAI research has advanced the concept of latent factor modeling, where a generative model can be used to perform inference on a low-dimensional set of unobservable quantities representing the underlying causes of measured data. Using this perspective, Rozzell’s group have developed an accurate and generalizable brain biomarker for recovery during subcallosal cingulate cortex (SCC) deep brain stimulation (DBS) for treatment-resistant depression (TRD). This data-driven biomarker has been shown accurate at capturing meaningful clinical outcomes, generalizes to new patient populations, and shows promise in case studies which have demonstrated clinical decision making. This biomarker is an objective anchor of a brain state that can help us shed light on the microcircuit structures and complex behaviors that underly treatment resistant depression pathology and recovery.

## Movement disorders

### From the lab to the home—towards naturalistic adaptive DBS for Parkinson’s disease

There has now been a decade of human adaptive DBS (aDBS) studies performed in Parkinson’s disease (PD) populations, which have provided proof-of-principle that tracking neural biomarkers and adjusting stimulation may improve clinical efficacy and reduce symptoms. However, these studies have previously been brief in duration, collected during the post-operative period and experiments have been performed in a single medication state with comparison to only partially optimized conventional DBS. Simon Little’s group presents data from a real-world, blinded, aDBS study in Parkinson’s patients (n = 4) (Oehrn et al., 2024). This approach was personalized to patients’ individualized symptoms and used machine learning to identify the optimal neural biomarkers for tracking symptom fluctuations in the clinic and at home, in the ON-medication state. The data pipeline converged on stimulation-entrained gamma oscillations (65 Hz—half stimulation frequency) that were present in the cortex (6 hemispheres) or subthalamic nucleus (STN) (3 hemispheres), as a highly robust biomarker of ON-medication states. The designed algorithm reduced the stimulation amplitude in the ON-medication state and increased the stimulation amplitude in the OFF-medication state. This resulted in a ~ 50% reduction in the time with the most bothersome symptoms (bradykinesia or dystonia) and improved quality of life. Overnight, the gamma biomarker was reduced, resulting in higher levels of night-time stimulation and better maintenance of sleep quality. This algorithm, however, wasn’t designed to optimize sleep, and Little’s group also presented data from multi-night at-home streaming of cortico-basal neural signals during sleep. This data revealed that subcortical beta oscillations are inversely correlated with healthy cortical slow waves during NREM and increase prior to spontaneous awakenings (Anjum et al., 2024). A classification algorithm using intracranial signals was used to detect awakening events on a much faster time scale (5 s) than classical (30 s) sleep staging. Provisional evidence from sleep-stage targeted aDBS suggests that this could support increased slow wave activity (Smyth et al., 2023). Overall, real-world validation of aDBS is moving out of the laboratory and into the home, and this marks a potential transition point towards widespread clinical application. Further validation work is required with larger numbers of subjects, as well as improved simplicity and automation of programming, to ensure that aDBS is scalable. Additionally, as daytime motor aDBS shows success and promise, there is now an opportunity to develop aDBS for non-motor symptoms of PD inclusive of sleep.

### Stimulation evoked resonant neural activity in the subthalamic nucleus is modulated by sleep and outperforms spectral features in decoding sleep

Sleep disturbances, including fragmented sleep and insomnia, are common in PD (Zahed et al., 2021). DBS of the STN is an effective therapy for PD, but DBS settings that are tuned to improve day-time motor function and deal with daytime motor fluctuations may not be optimal for sleep. Recent studies, based on one single case, suggest that reducing stimulation intensity during non-rapid eye movement (NREM) sleep may increase slow waves during sleep (Smyth et al., 2023). In addition, beta-triggered adaptive DBS may need to adjust the beta threshold to capture pathological activities during NREM sleep, as average beta power is reduced during NREM sleep (Thompson et al., 2018; Yin et al., 2023; Zhang et al., 2024). These studies highlight the importance of decoding sleep stages to further improve the efficacy of DBS delivered during sleep. DBS evokes resonant neural activity (ERNA) in the STN and globus pallidus internus (GPi) (Johnson et al., 2023; Sinclair et al., 2018). This oscillatory response to stimulation has an especially prominent amplitude in the dorsal subregion of the STN, and is associated with clinical outcomes (Steiner et al., 2024). It is also a promising biomarker for lead localization and tailoring stimulation parameters (Thevathasan et al., 2020). Huling Tan’s group reports for the first time that ERNA tracks sleep onset and sleep stage transitions (Wiest et al., 2024). Given the heterogeneity of beta power during sleep (De Hemptinne et al., 2023), its susceptibility to movement artifacts and superior classification accuracy of models using ERNA features, this finding paves the way for ERNA as a marker for automatic stimulation titration during sleep and for improved patient care.

### Novel approaches to adaptive DBS in Parkinson disease

Dennis Turner’s group has focused on fully implementing the Medtronic Summit RC + S capabilities for full external control approaches in a clinical trial of dual STN + GPi DBS in PD. After recruiting 6 patients with DBS-eligible advanced Parkinson disease symptoms, they implanted bilateral electrodes in both typical treatment locations (STN and GPi) with connection to a single RC + S implanted pulse generator (IPG) (Mitchell et al., 2022). The single IPG facilitated synchronization of all 4 output channels from the electrodes for analysis of phase locking and coherence. Using the streaming output from the electrodes, they have developed a complex control program for aDBS on a PC, initially using a proportional-integral (PI) controller based on beta band power, to update the DBS output values (Schmidt et al., 2024). The expected clinical outcome has been confirmed at 2 years as well as confirmation of a synergistic balance of symptom control with combined stimulation of STN + GP, with suppression of dyskinesias as well as tremor, which was also associated with significant medication reduction. As a result of this medication reduction and lack of dyskinesias, gamma frequency responses (typical of medication-induced dyskinesias) were not present in these patients. Through implementing the PI aDBS control program, they achieved similar clinical results, but reduced power usage across the patients. Potential benefits of STN + GP aDBS on speech and gait remain to be quantified using this paradigm, but previous work in STN aDBS suggested potential benefit (Little et al., 2016) in line with reducing the total electrical energy delivered. Further, Turner’s group developed a reinforcement learning (RL) controller for a fully automated controller parameter designed to predict control of DBS amplitude during dynamic activity as well as to enhance symptom control (Gao et al., 2023). Such RL controllers may be trained offline from existing data which is agnostic to controller type (i.e., previous RL iterations or PI controller). They evaluated the clinical relevance of both physiological biomarkers (i.e., beta band power, coherence) and external biomarkers using a random amplitude interrogation which was referenced to a clinical-behavioral measure. Turner anticipates that there will be translation of many of these features into available in an external control format and into a hierarchical, nested control format, taking advantage of both the simpler internal control format in combination with external signal integration.

## Stroke, traumatic brain injury, and sleep

### Chronic intracranial recordings to study circadian rhythms in movement disorders

**Figure 1:**
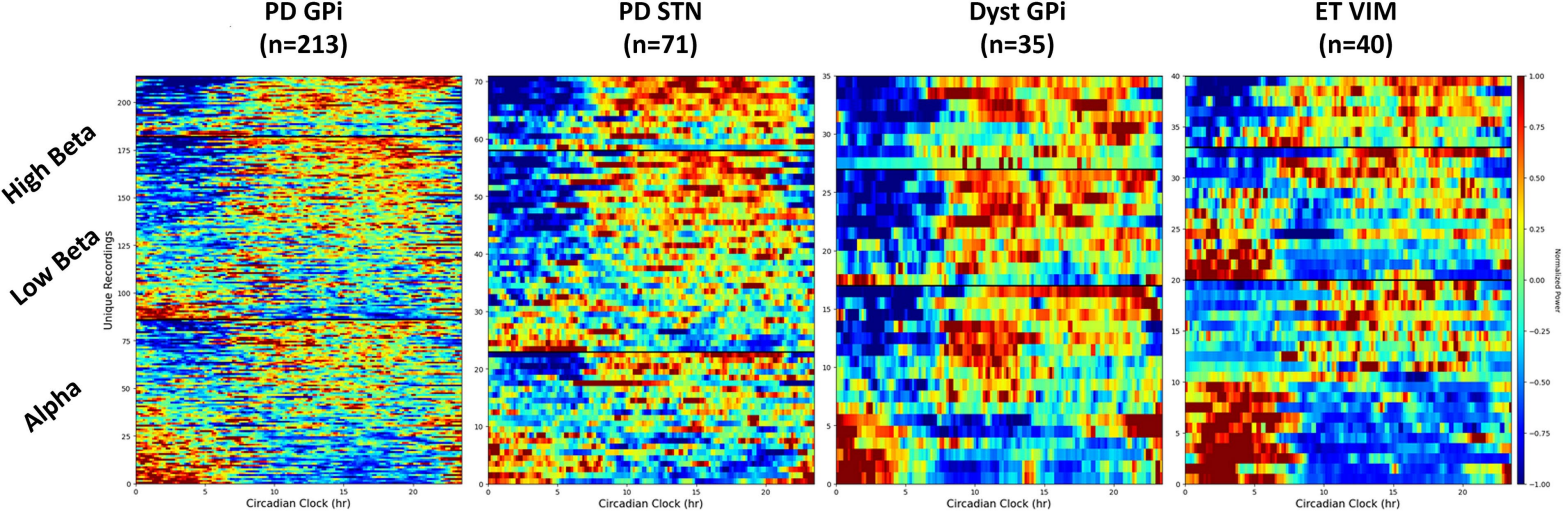
Spectrogram of LFP in different frequency bands throughout the day. Different circadian patterns of increased activity within a specific frequency band are seen across different conditions and targets. Dyst, dystonia; ET, essential tremor; GPi, globus pallidus pars interna; PD, Parkinson’s disease; STN, subthalamic nucleus.

Circadian rhythms have been observed in the STN of individuals with PD, however research on the GPi remains limited. This retrospective study examined GPi circadian rhythms in a large cohort of 93 subjects with PD, and a total of 130 recordings collected chronically in their home settings. A significant difference in GPi activity between day and night was uncovered in the majority of participants. Although most recordings showed a decrease in activity at night, an increase in power at night was observed in 26%. Reduction of power at night was more commonly found in higher frequency bands (above 20 Hz) and in patients on extended-release levodopa. These results suggested that circadian variations in GPi activity differed among individuals, with night-time increases potentially indicating the return of abnormal neural patterns. A similar trend was found in the STN of PD patients (n = 72), with STN beta power increased at night in 14% of the recordings. This contrasted with recording in the GPi of dystonia patients (n = 35) showing a reduction in beta power in all but one recording. Low beta power (13–20 Hz) in the ventralis intermediate nucleus (VIM) of the thalamus in patients with essential tremor (n = 40) was equally increased or decreased at night, but high beta power (20–30 Hz) was always reduced at night (Figure 1). Circadian rhythms in the alpha band (≤ 12 Hz) were similar across diseases and targets and could either increase or decrease at night (~50%). Understanding these fluctuations will be essential for the successful application of adaptive deep brain stimulation strategies in the real-world.

### DBS for post stroke rehabilitation: a translational project

Andre Machado’s group investigated the physiology of cerebello-thalamo-cortical pathways for the past two decades, initially with a focus on acquired brain injury (stroke and trauma), followed by opportunities to alleviate movement disorders that are typically refractory to traditional pharmacological and surgical modalities.

**Figure 2:**
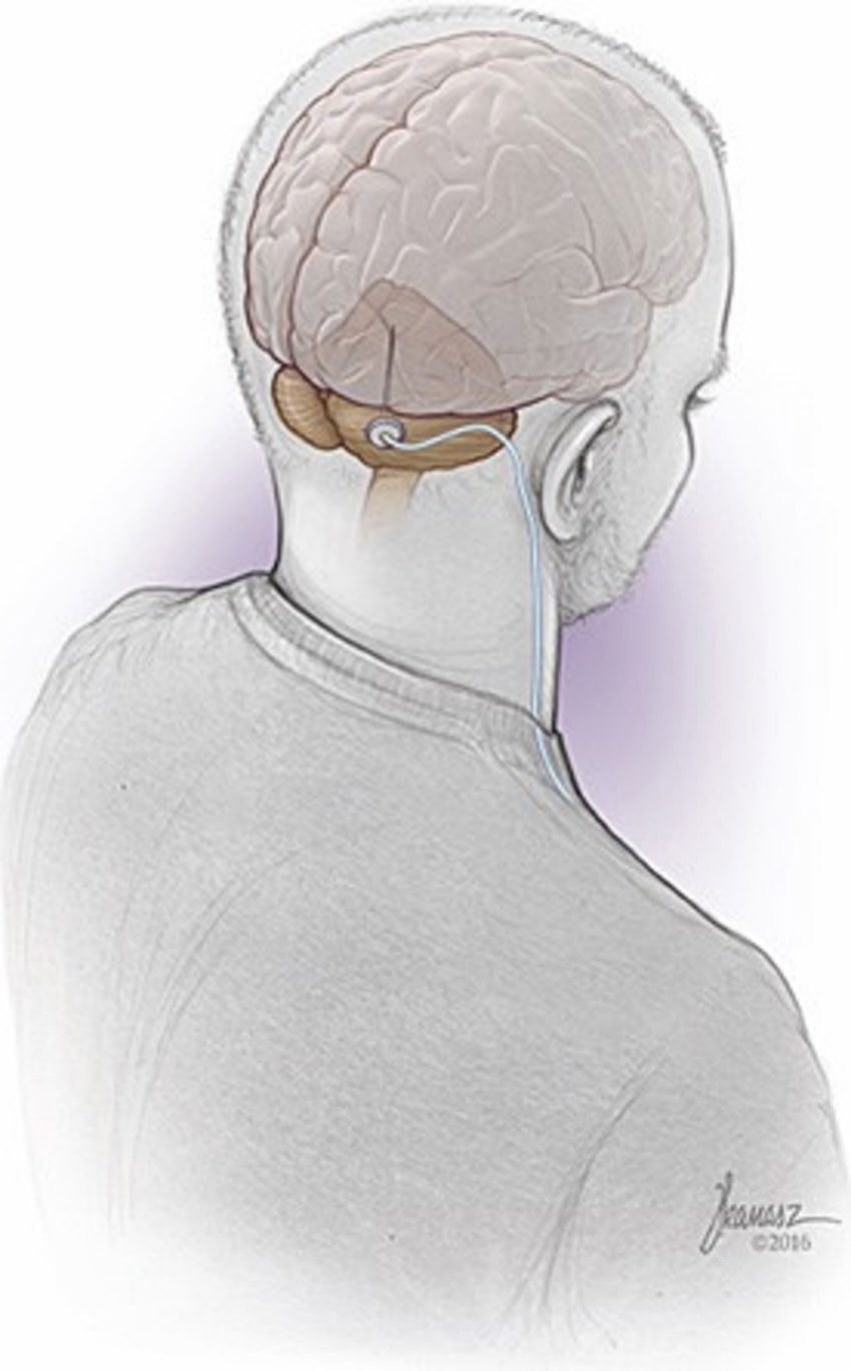
Illustration of a DBS lead in the right cerebellum.

Machado has shown, to date, that DBS targeting the dentate nucleus of the cerebellum (Figure 2), or its anatomical equivalent in the rodent model, modulates cortical excitability in the normal state, as well as in the post-stroke perilesional cortex of rodents and patients. Stimulation is associated with increments in expression of markers of perilesional plasticity, and of long-term potentiation, and also a doubling of perilesional synapses.

They have recently concluded a Phase I clinical trial of dentate nucleus DBS in a group of 12 patients with severe or moderate-to-severe post-stroke hemiparesis. Participants achieved a motor gain plateau after 3–5 months of supervised rehabilitation before the DBS was activated, followed by clinically and statistically significant gains when DBS was combined with rehabilitation. These gains were linearly correlated with increments in perilesional glucose metabolism. Machado’s group are currently conducting the first randomized controlled study of DBS for post-stroke rehabilitation.

### Central lateral thalamic nucleus/dorsal tegmental track medial deep brain stimulation improve chronically impaired executive function in moderate to severe traumatic brain injury

Patients with moderate to severe traumatic brain injury (msTBI) commonly suffer enduring cognitive impairment, typical impaired executive attention limiting their ability to focus, stay on tasks, and successfully reengage in work and educational activities. Successful treatment of impaired mental processing speed and executive function could improve patient quality of life. Nicholas Schiff’s group recently completed a 6-participant feasibility study (CENTURY-S, NCT02881151, funded by the National Institutes of Health BRAIN Initiative grant UH3 NS095554) of central lateral thalamic nucleus/dorsal tegmental track medial deep brain stimulation (CL/DTTm DBS) therapy in msTBI. The proposed underlying mechanism for the application of CL/DTTm DBS was to activate frontostriatal systems that were chronically downregulated by deafferentation. Converging evidence from prior work in rodents, non-human primates and humans demonstrated improved arousal and executive function through stimulation of the contralateral nucleus (CL). Schiff pre-selected a primary outcome measure, the Trail Making Test Part B (TMT-B) as an index of executive function and information processing speed, choosing a threshold of 10% improvement as a success. Six patients underwent successful implantation of bilateral electrodes into the CL, specifically targeting the medial dorsal tegmental tract (DTTm) guided by diffusion tensor imaging tractography and a participant-specific map of the thalamus generated by the Thalamus Optimized Multi Atlas Segmentation (THOMAS) pipeline (Su et al., 2019; Tourdias et al., 2014). Five participants with longstanding functional disability related to persistent cognitive dysfunction after severe TBI (age 23–60, 3–18 years after injury) completed the treatment phase of the study; one successfully implanted participant was withdrawn a month following surgery due to scalp infection.

All 5 participants completing the treatment phase met the pre-selected primary outcome benchmark of a greater than 10% improvement in completion time on the TMT-B from pre-surgical baseline to the end of the trial period (median = 31.75%; range 15–52%). Improvement in processing speed was observed on both the TMT-B measure and the related TMT-A measure. These changes were concordant with the self-reported improvements noted on the Traumatic Brain Injury Quality of Life-Attention measure (average improvement 79.5%). Despite the short 3-month open label phase, two of the four subjects who completed the trial showed a 1-point increase in the Glasgow Outcome Scale-Extended (GOS-E) rating from the presurgical baseline to the end of the trial period. These findings demonstrated the safety of implantation and initial demonstrations of improved mental control under speeded conditions in chronic msTBI. As shown in Table 1, the improvements in TMT-B and TMT-A were broad across the participants with similar overall effect size, despite a wide range of starting performance levels. Demographically adjusted T-scores showed that on average both measures were moved a full standard deviation across the population distribution (one subject had no demographically adjusted change in T-score on TMT-B, all others showed this effect size on both).

**Table 1:** Pre- and post-trial results of TMT and related TBI QoL attention and executive function self-report measures for each participant.

Participant	1	3	4	5	6	Mean
TMT—raw scores						
Part B						
Pre-surgery baseline	153.0	171.7	39.0	42.6	166.6	114.6
Treatment end	129.7	82.9	29.0	32.4	96.2	74.0
Percent change	−15.2	−51.7	−25.6	−23.9	−42.3	−31.8
Part A						
Pre-surgery baseline	62.0	85.9	22.1	18.7	61.6	50.1
Treatment end	41.5	45.9	16.5	14.7	44.7	32.7
Percent change	−33.1	−46.6	−25.3	−21.4	−27.4	−30.8
TMT—demographically adjusted T-scores						
Part B						
Pre-surgery baseline	22	33	62	62	22	40.2
Treatment end	22	50	71	71	35	49.8
Change over time	0	17	9	9	13	9.6
Part A						
Pre-surgery baseline	21	20	49	57	22	33.8
Treatment end	30	38	66	71	31	47.2
Change over time	9	18	17	14	9	13.4
QoL Attention						
Pre-surgery baseline	19	6	14	10	14	12.6
Treatment end	30	10	21	23	27	22.2
Percent change	58	67	50	130	93	79.5
QoL Executive Function						
Pre-surgery baseline	36	20	25	28	24	26.6
Treatment end	43	20	32	43	39	35.4
Percent change	19	0	28	54	63	32.7

CL/DTTm DBS aims to compensate for the widely deafferented substrate of anterior forebrain function underlying chronic cognitive impairment in msTBI (Schiff et al., 2023). The CL nucleus is both anatomically and physiologically specialized to support arousal regulation by providing strong synaptic drive across the frontal and prefrontal cortices and the rostral striatum in response to cognitive demands that support these “executive functions” (Baker et al., 2016; Liu et al., 2015). Schiff’s group proposes that restoration of integrative function of these neurons is supported by increased rates of background synaptic activity which are known to increase the dynamic range of information processing via improving spatiotemporal integration of inputs across the neuron. Such effects are likely most prominent within layer V cortical neurons of the frontal cortices (Schiff, 2020).

Electrical stimulation of the primary CL lateral wing cell bodies/DTTm fibers was not associated with abnormal sensations or movements. Regions more ventral to CL/DTTm elicited transient side effects in patients including speech slurring, jaw sensations and perseveration; these effects may relate to activation of the centromedian-parafasicularis and more medial components of the median dorsalis nucleus, respectively.

This is the first study of DBS electrode implantation in moderate to severe traumatic brain injury with subsequent recovery (outcome range of GOS-E 5 to 7) to remediate impaired cognitive function. The generalizability of these findings will require testing in a larger sample.

## Advances in commercially available neuromodulation technology

The Think Tank hosted scientific presentations from industries that are leading in neuromodulation technology. These included not only the leading manufacturers of DBS devices, but also novel neuromodulation technologies.

### Medtronic

While DBS provides an effective therapy for a number of approved indications, industry expansion into novel indications has been limited, despite widespread interest in both novel disorders and stimulation targets (Harmsen et al., 2021). Previous industry-sponsored research and pivotal trials for such indications as treatment resistant depression and Alzheimer’s disease, have shown promise, but ultimately left the field with more questions on how to approach the development of new approved DBS therapies. An intentional and systematic approach to trial design, biomarker discovery, parameter optimization, objective outcome measures, and market assessment will be critical for advancing new therapy concepts into commercial access.

A recent explosion of neurotechnology and data access has provided a significant opportunity to enable new therapy development using data-driven insights. In particular, neural sensing provides novel visibility into objective, patient-specific biofeedback that has the potential to guide where, when, and how to deliver stimulation therapy. New commercially available neuromodulation technology platforms, such as Medtronic Percept™ devices with BrainSense™ allow chronic sensing of brain signals from DBS leads while delivering stimulation both in and out of the clinic. Since the Percept market release in 2020, this capability has been available in tens of thousands of DBS patients worldwide. Using BrainSense technology, there has been growing evidence that patient-specific physiological feedback can be used to streamline initial DBS programming (Binder et al., 2023; Lewis et al., 2024; Swinnen et al., 2023) and to guide optimization (Feldmann et al., 2021; Vaou et al., 2023) in people with Parkinson’s disease. The same principles of using objective biofeedback to guide therapy decisions are emerging as a critical component in therapy development for other approved indications as well as for indications currently under investigational status. Research using a combination of approved and investigational Medtronic DBS systems has consistently demonstrated that chronic brain sensing can provide a method of symptom or response tracking in people with treatment resistant depression (Alagapan et al., 2023), Tourette’s syndrome (Shute et al., 2016), epilepsy (Gregg et al., 2021), and obsessive compulsive disorder (Provenza et al., 2024). Embedded brain sensing technology will be key in enabling biomarker discovery, classifier development, and uncovering potential mechanisms of action needed for tailoring DBS in a disease-specific and personalized fashion.

As neurotechnology to support chronic monitoring of patient states emerges, users will have access to more data than ever before. Fundamentally, it is critical that data provide clinically meaningful insights to ultimately make therapy programming and optimization easier or to produce better outcomes for the patient. For example, neural sensing has been used to automate electrode selection, reducing the time spent during an initial programming session (Binder et al., 2023; Thompson et al., 2023). Furthermore, aDBS may alleviate the patient burden by moving towards a more personalized and automated therapy delivery with potentially better clinical outcomes (Oehrn et al., 2024). The Medtronic-sponsored ADAPT PD trial (Bronte-Stewart et al., 2023), which automatically adjusts stimulation amplitude based on a biomarker of patient state, is a critical trial for providing access to more automated therapy. As automated features continue to be developed across therapies, Percept is an example platform that has been intentionally designed to be software and firmware upgradable.

### Boston Scientific

The success of DBS therapy hinges on accurate lead positioning and optimal stimulation programming, which often requires the clinician’s expertise to test various parameters commonly through trial and error. Image Guided programming (IGP) pairs visual interaction of neuroimaging data (patient-specific anatomy or aggregate priors) with 3D lead objects and stimulation field models to reduce programming time (56–75%) while achieving similar outcomes (Lange et al., 2021; Waldthaler et al., 2021, p. 2).

Boston Scientific is currently advancing these DBS workflows via commercially available platforms GUIDE XT and STIMVIEW XT. To further enhance programming, Boston Scientific has been working on the DBS Illumina 3D (I3D) algorithm that automates the initial search process (Malekmohammadi et al., 2022). This algorithm identifies stimulation parameter sets that maximize therapeutic region stimulation while minimizing unintended stimulation of side-effect regions. With designed flexibility integrated into the algorithm, the settings proposed by DBS I3D serve as a suitable starting point for exploring the treatment space.

Boston Scientific is conducting a clinical study to gather data on the DBS I3D algorithm and to answer critical questions as to what to and what not to stimulate, and how to more efficiently stimulate specific brain areas.

This ongoing prospective study selected patients who were previously implanted with a DBS system in the bilateral STN and had a stable (at least 4 weeks), optimized standard of care DBS program. During a single study visit, motor symptoms were evaluated in a medication off/DBS off condition, followed by comparing the patient’s standard of care program to the initial program derived by the DBS I3D algorithm by a blinded assessor. Once the patients were in their med on state, the algorithm-derived program was further optimized by the treating neurologist and evaluated by the blinded assessor (medication on/DBS on).

Boston Scientific presented the preliminary findings that (1) initial stimulation settings suggested by the algorithm produced statistically comparable motor scores to the optimized standard of care settings (medication off/DBS on), in a matter of seconds as opposed to the weeks and months required to derive the optimized standard of care setting. (2) Statistically significant improvement was observed in the motor scores (medication on/DBS on) using the optimized settings derived by the DBS I3D algorithm compared to baseline (medication off/DBS off). (3) The optimized stimulation settings (i.e., the send home settings) were similar to the initial stimulation settings suggested by the algorithm.

### Abbott

Innovation alone is insufficient to address global health challenges without ensuring access to technology. Current patient needs for DBS therapies include (1) therapy awareness, (2) simplicity of use, and (3) care access. Abbott aims to address these needs by focusing on innovative technology, piloted through clinical feasibility studies and those focused on improving clinical outcomes.

Abbott’s recent LibertaRC™ platform minimizes complexity for patients while offering an avenue to advance research into DBS therapy. Specifically, with an emphasis on patient centricity, Abbott devised a platform optimized for size and charging burden. Coupled with these features is an enhancement to Neurosphere™ Virtual Clinic, their remote programming platform focused on improving care-access, incorporating features like live remote monitoring of IPG charging. In this platform, Abbott built out the capability to expand the digital ecosystem to facilitate rapid innovation. For example, the platform can be configured for research to support multiple behavioral sensors. Similarly, this platform has upgradeable capabilities like the waveform player that can be leveraged into the future development of custom waveforms. This approach aims to extend DBS applications to new targets and indications, ensuring broader access and increased therapy awareness.

These technical advances seamlessly integrate into their clinical research pipeline. The ROAM-DBS study leveraged a decentralized platform for remote data collection, which improved the granularity of their data while maintaining privacy and cybersecurity standards. The study demonstrated that patients with Virtual Clinic access achieved improved outcomes faster than those without (Tomlinson et al., 2023).

To continue increasing care-access by providing therapy options for patients who could benefit from it, Abbott is addressing treatment-resistant depression with DBS, following a breakthrough designation in 2022. A pivotal multicenter randomized controlled trial will enroll 100 patients across up to 25 U.S. sites. The primary endpoint is focused on depression symptom reduction measured by the Montgomery–Åsberg Depression Rating Scale at 12 months. Key factors considered in the study design include response time, patient profile, and target selection within the subgenual cingulate cortex.

In conclusion, Abbott is committed to bridging gaps in DBS therapy by driving innovation aimed at expanding access and improving outcomes in order to better serve patients.

### NeuroPace

The NAUTILUS Study (NCT05147571) is a pivotal clinical study to determine if the responsive neurostimulation for seizures (RNS) system is safe and effective as an adjunctive therapy for the treatment of primary generalized seizures in individuals 12 years and older who have drug-resistant idiopathic generalized epilepsy (IGE). This prospective, multicenter, single-blind, randomized, sham stimulation-controlled study has enrolled 100 participants within the United States. Leads were placed bilaterally in the centromedian nuclei of the thalamus. Primary outcome measures are the 12-week post-operative serious device-related adverse event rate and the time to second generalized tonic–clonic seizure.

The RNS System Lennox–Gastaut Syndrome (LGS) Feasibility Study (NCT05339126) is an National Institute of Neurological Disorders and Stroke funded Brain Initiative study intended to generate preliminary safety and effectiveness data for brain-responsive neurostimulation of thalamocortical networks as an adjunctive therapy in reducing the frequency of generalized seizures in patients 12 years or older with LGS who are refractory to antiseizure medications (Warren et al., 2024). The study has enrolled all 20 subjects. Leads are placed bilaterally in pre-frontal cortex and centromedian nuclei of the thalamus.

NeuroPace research has validated a deep learning model to sort chronic ECoG data. The model has demonstrated non-inferiority to human reviewers (Arcot Desai et al., 2023). NeuroPace has also demonstrated in a cohort of ~4,000 patients that the majority have multidien seizure rhythms that an advanced machine learning model can use to forecast rhythms over the next 30 days (Norman et al., 2024).

### Newronika

AlphaDBS is an implantable closed-loop deep—brain stimulation system. The system features advanced filtering technology for detecting LFP sensed through the DBS lead. The implanted stimulator also utilizes a linear control algorithm that adjusts stimulation parameters according to the power in a selectable frequency band of a LFP. The system has 16 independently controlled stimulation and two sensing channels, one per hemisphere. Via a telemetry unit and a patient app, LFP data recorded 24/7 can be uploaded to a cloud-based database, with no data loss or overwriting. The system is compatible with octopolar DBS leads (Medtronic 3389 leads) and with directional leads (Abbott directional leads).

The fully implantable system has received CE-Mark for conventional DBS plus sensing for the treatment of PD, but not for adaptive DBS.

**Figure 3:**
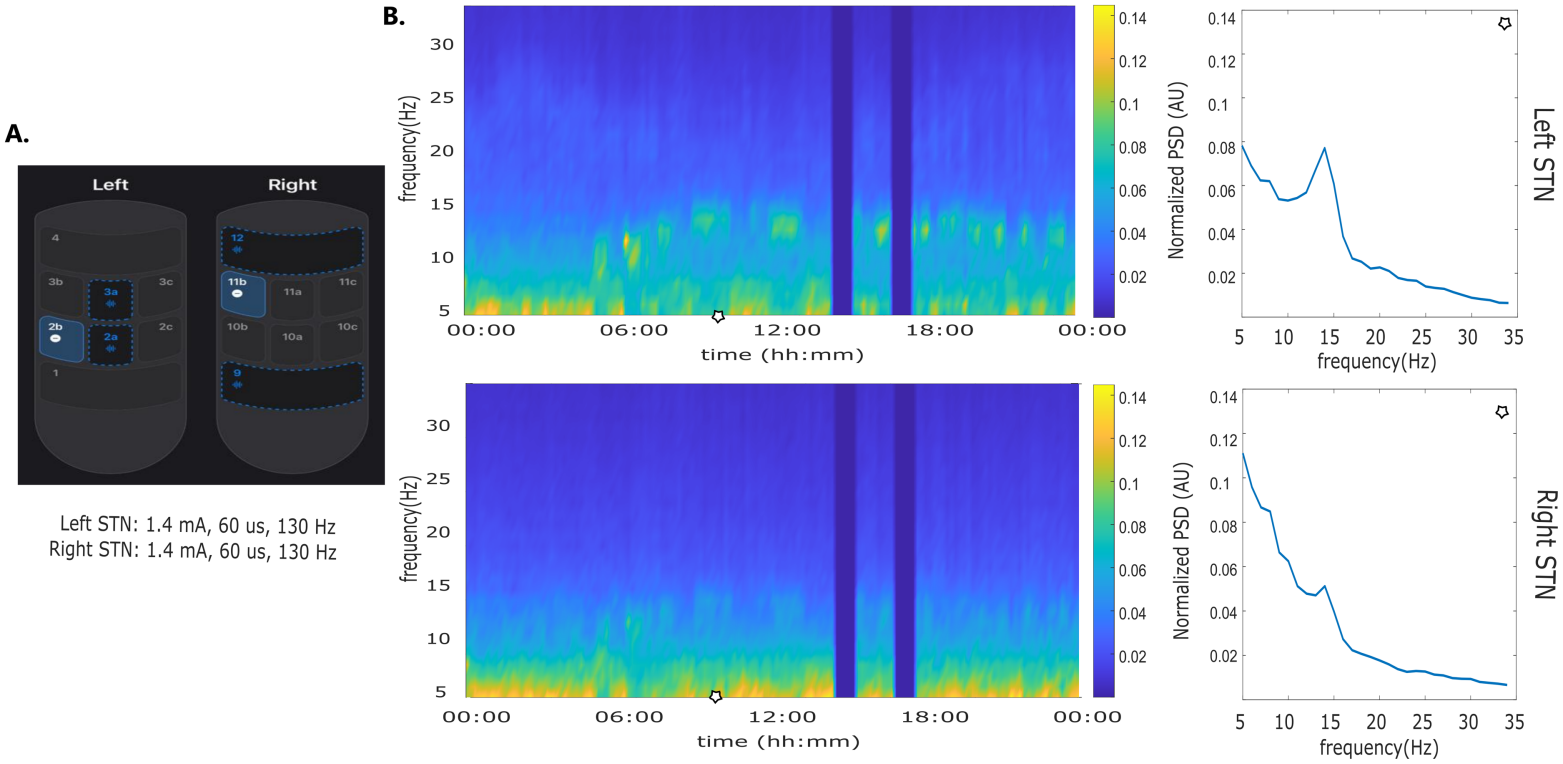
Asymmetric sensing with stimulation on. **(A)** Stimulation and sensing configuration: Left STN stimulation contact 2b, sensing contacts 2a and 2a; Right STN stimulation contact 11b, sensing contacts 9 and 12. Sensing contacts 9 and 12 and not at equidistance to contact 11b. **(B)** time frequency plot and normalized amplitude spectrum calculated at time points indicated by the blue line.

The use of adaptive investigational DBS devices has been described with LFP recording in the so called symmetrical or “sandwich” configuration with recording contacts at equidistance on opposite sides of the stimulation electrode (Gilron et al., 2021; Stanslaski et al., 2012). The AlphaDBS system, can reliably detect LFP in both the symmetrical and asymmetrical sensing configuration with stimulation “on” (Arlotti et al., 2021) (Figure 3). This configuration facilitates more flexibility in setting aDBS contact configurations, to further explore the pathophysiology and DBS network effects by increasing not only the stimulation, but also the sensing specificity. In a recent feasibility study, PD patients were implanted with an AlphaDBS system using Abbott directional leads placed in the subthalamic nucleus. The greatest beta activity was found in the contacts closest to the stimulation sites in an asymmetric position, which have also been reliably used for aDBS delivery.

### Motif

Motif is developing a miniature epidural cortical stimulation implant that targets the transcranial magnetic stimulation (TMS) site for treatment of depression. This device is specifically designed for patients who initially respond to TMS but frequently relapse in the months following a response to therapy. Motif’s architecture, a millimeter-scale, battery-free implant, has demonstrated the ability to surpass the motor threshold when placed above the dura during intraoperative human testing. The device can generate sufficient stimulation to elicit motor responses, comparable to those achieved by TMS, which will likely be essential for therapeutic efficacy in treating major depressive disorder (Sonmez et al., 2019, p. 201).

The technology leverages wireless power transfer to energize the device, enabling it to deliver precise cortical stimulation without the need for an implanted battery or complex wiring. This minimally invasive approach reduces the risks associated with traditional neuromodulation devices, such as lead migration and infection. Motif is currently seeking regulatory approval to initiate its first human study with this chronically implanted miniature brain stimulator, aiming to provide long-lasting, accessible treatment for patients who suffer from depression, but do not achieve lasting relief from TMS.

Implantable neuromodulation technologies for psychiatric conditions, like the Motif device and others, provide opportunities to record brain data in a way that may support dosing and provide alerts in advance of relapse events. This combination of therapy and monitoring in a closed-loop system implantable device provides a “psychiatric brain-computer interface” platform that can shift toward more data-driven care. As data becomes more available, there are opportunities for companies in this space to work together to better route patients to the best form of therapy, whether it is the type of cortical stimulation described by Motif, or the type of DBS described in other trials. This presents a win-win opportunity for industry and patients, in that patients will be able to select treatment options with greater confidence that they will be a responder, which in turn will likely enhance the adoption of the technology.

## Emerging magnetoencephalography-DBS research

### DBS-evoked potentials: from cortical signatures to clinical programming

Magnetoencephalography (MEG) can be used to investigate cortical signatures of responses evoked by a DBS pulse (Supplementary Video S1). These responses are referred to as DBS-evoked cortical potentials (cEP). STN stimulation leads to short-latency cEP (2–10 ms) that originate from the antidromic activation of the hyperdirect pathway fibers (Ashby et al., 2001; Bahners et al., 2022; Miocinovic et al., 2018; Walker et al., 2012). At latencies of more than 20 ms cEP might be generated through the orthodromic activation of the basal ganglia-thalamo-cortical loop (Devergnas and Wichmann, 2011). Previous work has suggested cEP as non-invasive markers for clinical programming in the context of STN-DBS and PD (Irwin et al., 2020; Miocinovic et al., 2018; Peeters et al., 2023; Romeo et al., 2019; Walker et al., 2012) and more recently in persons with depression treated with SCC-DBS (Seas et al., 2024; Waters et al., 2018) and GPi-DBS in dystonia (Bhanpuri et al., 2014; Tisch et al., 2008). None of the studies in PD have established a relationship between cEP amplitudes and motor performance.

Bahners et al. set out to study this relationship in a cohort of 22 persons with PD and recorded accelerometry during clinical testing to extract objective markers of bradykinesia for several stimulation settings (Bahners et al., 2023; Spooner et al., 2023, 2024a,b). Afterwards, MEG was recorded with the same stimulation settings but a lower stimulation frequency. After the stimulation pulse, the cortical distribution of cEP (Supplementary Video S1) aligned with the basal ganglia-thalamo-cortical network (Bahners et al., 2023). When extracting the cEP amplitude from the motor cortex, they observed a relationship between cEP amplitude and motor performance throughout the time course of the cEP (Bahners et al., 2023; Spooner et al., 2024b). Additionally, the amplitude of the cEP indicated optimal directional contact orientations (Spooner et al., 2023). These results are an important prerequisite to developing a cEP-informed DBS programming approach. This methodology could potentially translate to other DBS targets like GPi and SCC (Bhanpuri et al., 2014; Seas et al., 2024).

### Using MEG to study cortico-subcortical oscillatory connectivity

**Figure 4:**
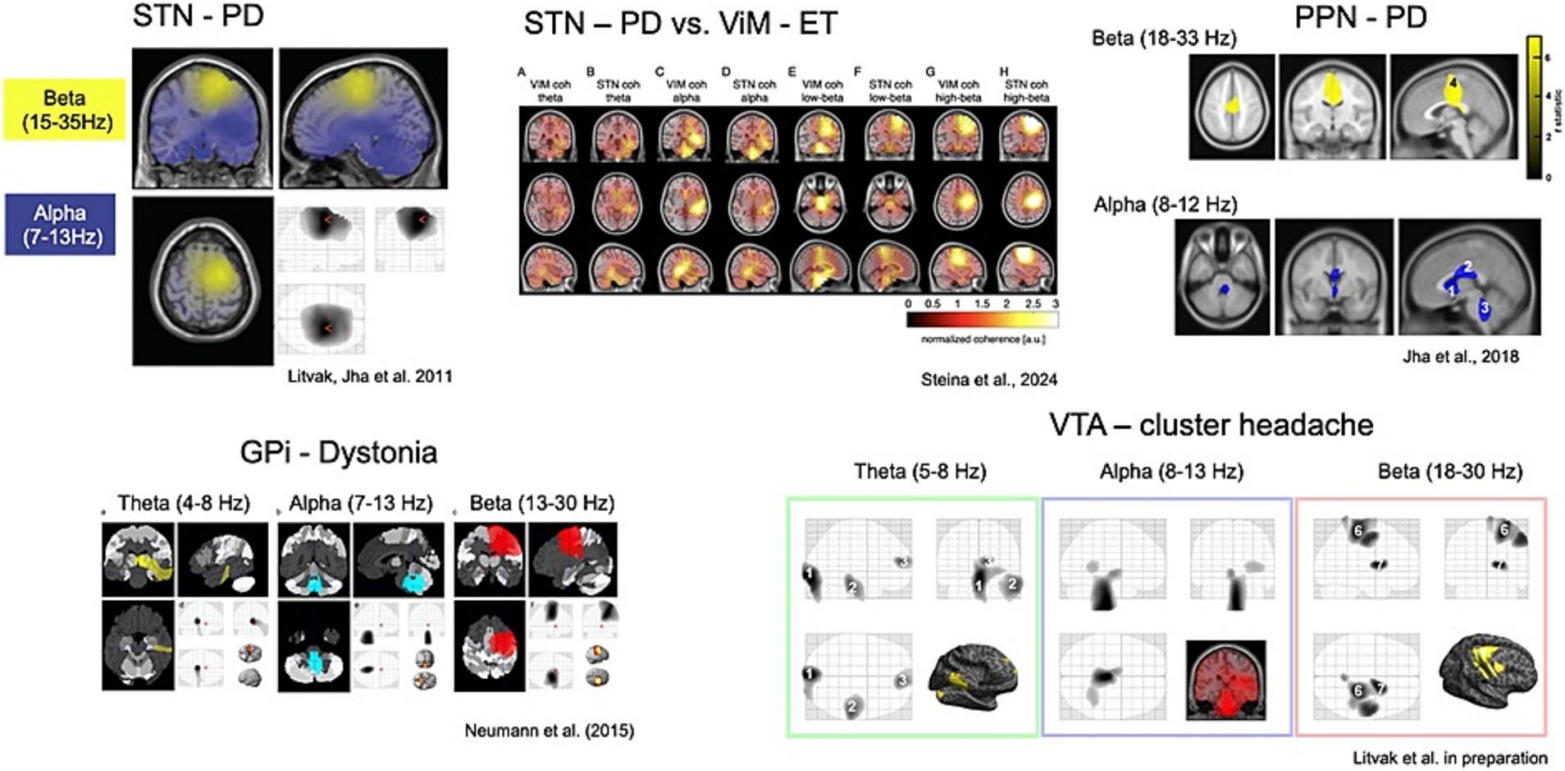
Oscillatory coherent networks of several DBS targets studied to date.

Combining invasive and non-invasive recordings in DBS patients offers a unique opportunity to study the dynamic interactions between DBS targets and the brain as a whole (Litvak et al., 2011, 2021) (Figure 4). Although more sensitive to artifacts generated by the implant and stimulation, MEG presents several advantages over electroencephalography (EEG) for mapping oscillatory phenomena and for differentiating activity from various brain sources.

By applying MEG source analysis to map the oscillatory coherence of subcortical targets, Vladimir Litvak’s group has characterized distinct frequency-specific networks, revealing both similarities and differences across targets and diseases (Litvak et al., 2021).

Their research demonstrated that cortico-subthalamic high beta coherence in Parkinson’s disease is distinct from pathological low beta activity observed in the basal ganglia and is associated with the hyperdirect pathway from the supplementary motor area (Swann et al., 2016; Oswal et al., 2021).

Narrow-band cortico-subthalamic high gamma coherence, initially identified using MEG, was linked to the high dopaminergic state and dyskinesias by subsequent studies (Swann et al., 2016).

Recently, new findings have linked the alpha-theta cortico-subthalamic network to cortical areas involved in auditory and speech processing, as well as to a potential subcortical pathway from the auditory brainstem to the STN (Jorge et al., 2022; Fahimi Hnazaee et al., 2024).

Future research directions include combining MEG with telemetric streaming in chronically implanted patients and harnessing the unique advantages of recently developed wearable MEG technology (Hnazaee et al., 2023).

### DBS for treatment resistant depression

DBS targeting the SCC has shown potential in treating treatment resistant depression (TRD) (Crowell et al., 2019). However, there are presently no readily evaluable clinical readouts to inform stimulation programming in real time.

As such, identifying functional biomarkers related to treatment success may expedite therapy optimization, in addition to contributing to an enhanced understanding of TRD and SCC-DBS mechanisms.

While others have utilized intracranial approaches for this purpose (Alagapan et al., 2023; Bijanki et al., 2022; Scangos et al., 2021), Dr. Luka Milosevic’s lab assessed MEG data from 7 SCC-DBS responders, 8 non-responders, and 25 healthy controls (Scherer et al., 2023). A statistical pipeline was developed to identify regions and networks of interest, based on oscillatory modulations that concurrently (i) distinguished people with TRD (with DBS off) from healthy controls and (ii) differentiated treatment responders from non-responders, based on responder-specific normalizations of this activity with DBS device in the on condition.

Numerous brain regions in the default mode, central executive, and somatomotor networks, including regions encompassing the dorsolateral and ventrolateral prefrontal cortices, were able to differentiate responders from non-responders based on decreases in the alpha band (8–12 Hz) hyperactivity and increases to gamma band (32–116 Hz) hypoactivity; congruent with recent stereo-EEG (sEEG) findings (Bijanki et al., 2022).

The identified biomarkers implicate key brain regions and networks involved in executive function, emotional regulation, self-referential thought, psychomotor function, social cognition, decision making, and other relevant functions. If these electrophysiological markers are able to serve as functional proxies for optimizing DBS therapy, MEG may represent an important, efficient, and non-invasive method for data-driven therapeutic guidance.

## Epilepsy

### Paradigm shift in epilepsy surgery: neuromodulation of thalamocortical circuits for the underserved epilepsy population

Medically refractory epilepsy (MRE) represents a public health crisis. Despite ample evidence of the futility in adding medication when two antiseizure medications have failed to control seizures, most patients continue to be treated medically, and epilepsy surgery remains grossly underutilized. In our experience, pediatric epilepsy surgery has informed the direction and utility of epilepsy surgery for the most difficult to treat network disorders associated with malformations of cortical development, epileptic encephalopathies, and other mutable epilepsy syndromes such as LGS and Tuberous Sclerosis.

**Figure 5:**
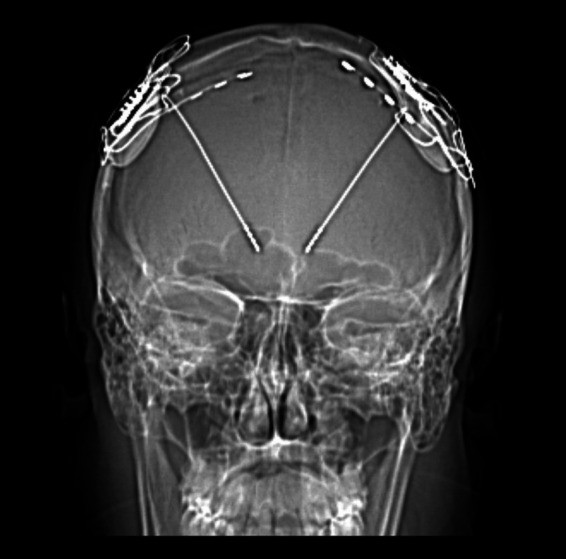
Frontal X-ray depicting DBS electrodes implanted in the bilateral thalamus, and neocortical strip electrodes.

Saadi Ghatan’s group has pioneered the use of DBS for treating these disorders in children and adults, predominantly with the use of the RNS, where closed loop thalamocortical neuromodulation is undertaken. Targeting anterior nucleus of the thalamus (ANT), centromedian (CM) and pulvinar nuclei, occasionally in conjunction with neocortical strip electrodes (Figure 5), Ghatan’s group has provided safe and progressively improved seizure control in greater than two thirds of patients. sEEG of thalamic nuclei at the time of intracranial electrode mapping has facilitated the use of individualized connectomics that may possibly offer better seizure control. A current randomized trial employing two RNS devices with frontal neocortical and CM electrodes aims to provide definitive evidence of the utility of a DBS approach to LGS. Recent publications that echo Ghatan’s group experience in the use of CM-RNS in generalized epilepsy, and the increased utilization of epilepsy surgery with neuromodulatory approaches in children, provide hope for using DBS strategies to treat MRE in people previously not considered candidates for surgery.

### A common brain circuit target for epilepsy?

Epilepsy is a brain network disease. Lesions are a common cause of epilepsy, but it has remained unclear what brain networks a lesion may trigger epilepsy. Recent advances facilitated mapping lesions to brain networks and identifying common brain circuits causally implicated in neuropsychiatric diseases, a technique termed ‘lesion network mapping’ (Boes et al., 2015). Combined with information from DBS, therapeutic relevance of these networks can be tested which is also called ‘DBS network mapping’ (Horn et al., 2017) and could identify a common brain circuit target for epilepsy (Fox, 2018).

**Figure 6:**
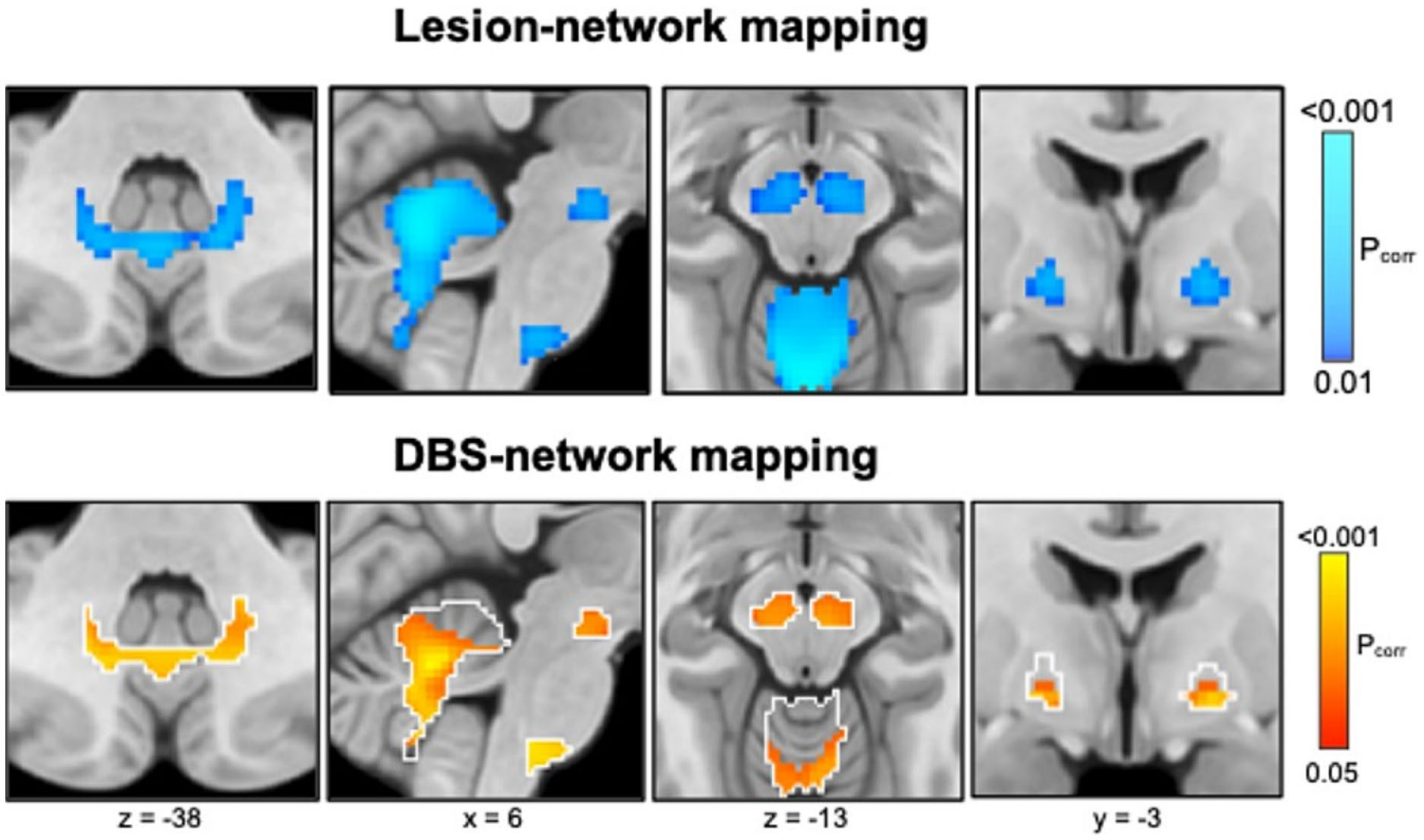
Lesion network mapping (top) identified that lesion locations associated with epilepsy were more negatively functionally connected (“anticorrelated,” cold colors) to the basal ganglia (globus pallidus internus, substantia nigra) and cerebellum. DBS network mapping (bottom) identified that DBS sites associated with better seizure control were more positively functionally connected (warm colors) to this same network (white outlines), converging on a common brain circuit target for epilepsy.

Schaper et al. (2023) studied 347 patients with lesion-related epilepsy and 1,126 patients with lesions but no epilepsy of different etiologies (strokes, hematomas, tumors, traumas, and tubers). Using lesion network mapping, they found that lesion locations associated with epilepsy were more functionally connected [“anticorrelated” (Fox et al., 2005)] to the basal ganglia (GPi, substantia nigra) and cerebellum (Figure 6, top panel), identifying a common brain circuit associated with lesion-related epilepsy. The therapeutic relevance of these connections was tested using DBS network mapping and data from 30 patients with anterior thalamic DBS for focal epilepsy. DBS sites associated with better seizure control were more functionally connected to this same brain network (Figure 6, bottom). The direction of functional connectivity of DBS was the inverse of lesions, consistent with the known scenario that lesions cause epilepsy while DBS treats epilepsy.

In summary, lesions causing epilepsy and DBS sites treating epilepsy converge on a common brain circuit for epilepsy which may offer an improved circuit target to guide brain stimulation.

### Thalamic stimulation induced changes in connectivity and epileptiform activity: insights from thalamic stereo-EEG

**Figure 7:**
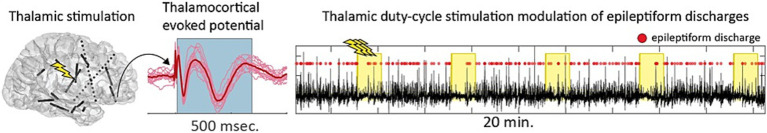
Single pulse and repetitive high-frequency stimulation of the thalamus during stereotactic electroencephalography (sEEG) are used to study the impact of thalamic neuromodulation on seizure network excitability.

Thalamic neuromodulation for epilepsy is limited by a lack of short-latency clinical signs or symptoms to guide stimulation parameter optimization, in contrast to DBS for other neurological disorders, such as essential tremor or PD. Seizure network location and extent is also highly patient specific, and optimal subcortical stimulation targets for a given individual are uncertain. Clinical sEEG including subcortical electrodes provides an opportunity to characterize cortical and subcortical seizure network nodes. A combination of single pulse electrical stimulation to map effective connectivity (directed causal influence between neural populations), passive recordings to track changes in interictal epileptiform discharges, and repetitive treatment stimulation, may provide new insights into the acute and subacute impact of thalamic stimulation on seizure network excitability and epileptiform abnormalities. Preliminary findings indicate that thalamic stimulation can modulate network interictal epileptiform activity and effective connectivity (Figure 7) (Gregg et al., 2024).

## Neuroethics in neuromodulation

### Translational neuroethics

Wexler and Specker Sullivan (2023) proposed a path forward for neuroethics based on the concept of translational neuroethics. They argued it is critical for neuroethical scholarship to move from theory to practice for the field to move forward. A translational neuroethics path requires integration and inclusivity. Integration is facilitated by having teams composed of neuroethicists, clinicians, and neuroscientists to help ground neuroethics scholarship in some of the compelling, current ethical challenges that are facing basic neuroscience and clinical researchers and, thereby avoid “speculative” neuroethics or ethics scholarship that prioritizes rare or unrealistic neuroscientific scenarios over more realistic, pertinent challenges. Inclusivity is achieved by examining who is engaged in the neuroethics scholarship, the questions asked, and incorporating careful considerations of how data arising from neuroethics scholarship and how it applies to a variety of different groups including patients.

Perhaps no topic illustrates the speculative nature of neuroethics more than the assertion that DBS results in unwanted, deleterious changes in personality and loss of control (Gilbert et al., 2021; Kubu et al., 2019). Empirical, prospective studies have demonstrated that DBS is associated with significant increases in patients’ perceptions of control (Kubu et al., 2017; Merner et al., 2021), greater manifestation of individually identified and valued personality characteristics so that patients feel they are closer to their pre-disease self, though there are minimal changes on standard personality tests. This prospective empirical data illustrate how a translational neuroethical approach can be used to improve informed consent processes and reduce the stigma associated with implanted neural devices (Merner and Kubu, 2023). Similarly, a translational neuroethics approach will be critical to identifying a viable path forward to address the vexing problem of device abandonment (Okun et al., 2024) and the tensions associated between the profound opportunities to understand basic brain mechanisms afforded by innovative neuromodulation versus the need to protect patients and the broader field (Fins et al., 2011). This approach requires humility and improved communication with all relevant stakeholders, so we avoid the mistakes of the past including paternalism.

### Why we need to define and address abandonment of DBS devices?

**Figure 8:**
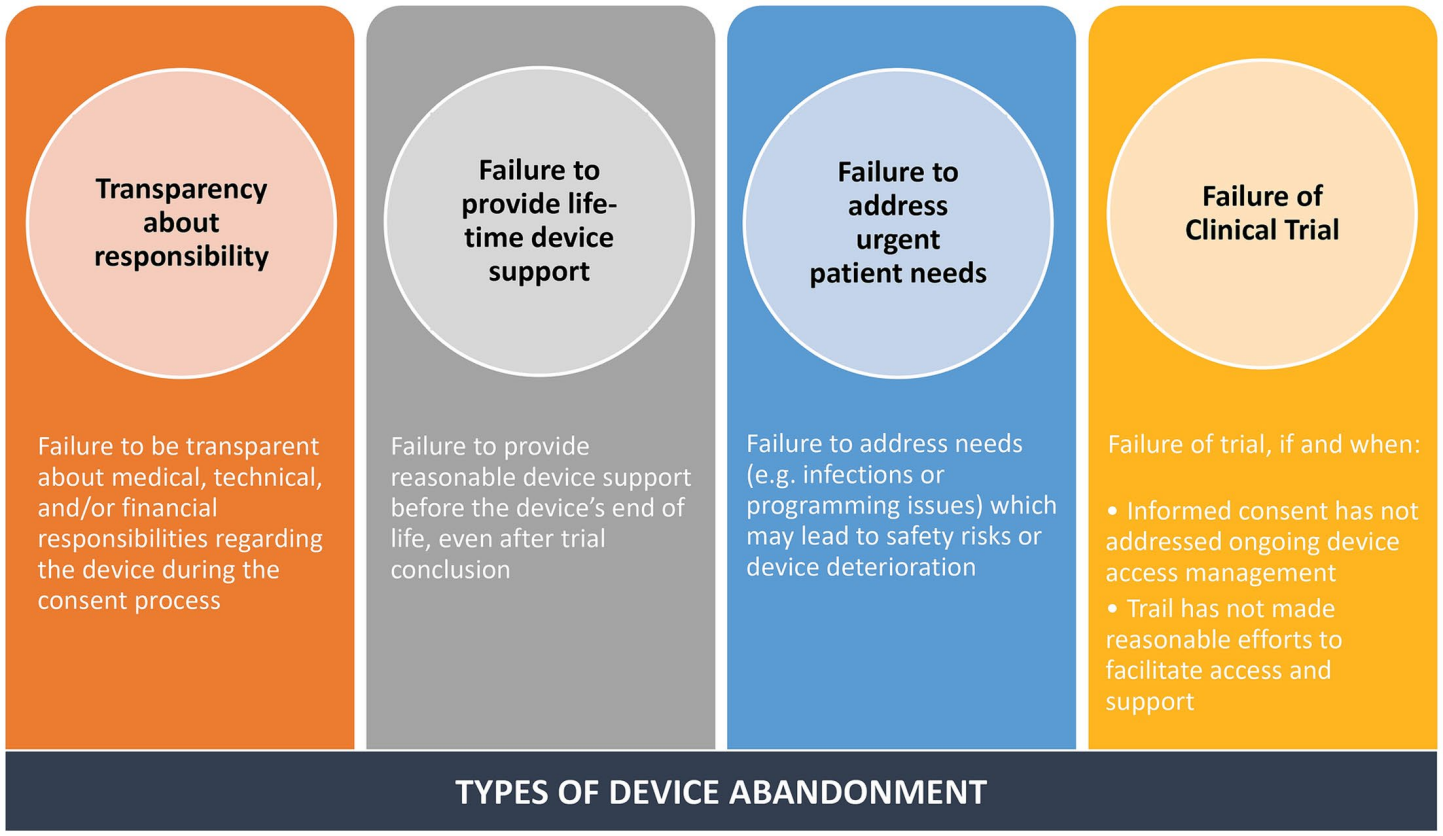
Types of device abandonment.

A critical oversight in DBS has been our lack of a definition for neurological device abandonment. Recently, Michael Okun’s group reviewed 734 articles published in the professional literature and found only 7 were relevant to address the issue of neurological device abandonment (Okun et al., 2024). They convened a multistakeholder group and developed a consensus definition for neurological device abandonment. DBS, vagal nerve stimulation, and spinal cord stimulation were all included in the definition. All stakeholders were included in the approach. Okun’s group suggest that the definition for neurological device abandonment includes failure (1) to provide fundamental aspects of patient consent, (2) to fulfill reasonable responsibility for medical, technical, or financial support prior to the end of the device’s labeled lifetime, and (3) to address any or all immediate needs that may result in safety concerns or device ineffectiveness. Finally, they also included a fourth point that the definition of abandonment associated with the failure of a research trial that should always be contingent on specific circumstances (see Figure 8).

### The untrivial nature of non-diagnostic brain biopsy during DBS: issues, implications, and imperatives for clinical practice

**Figure 9:**
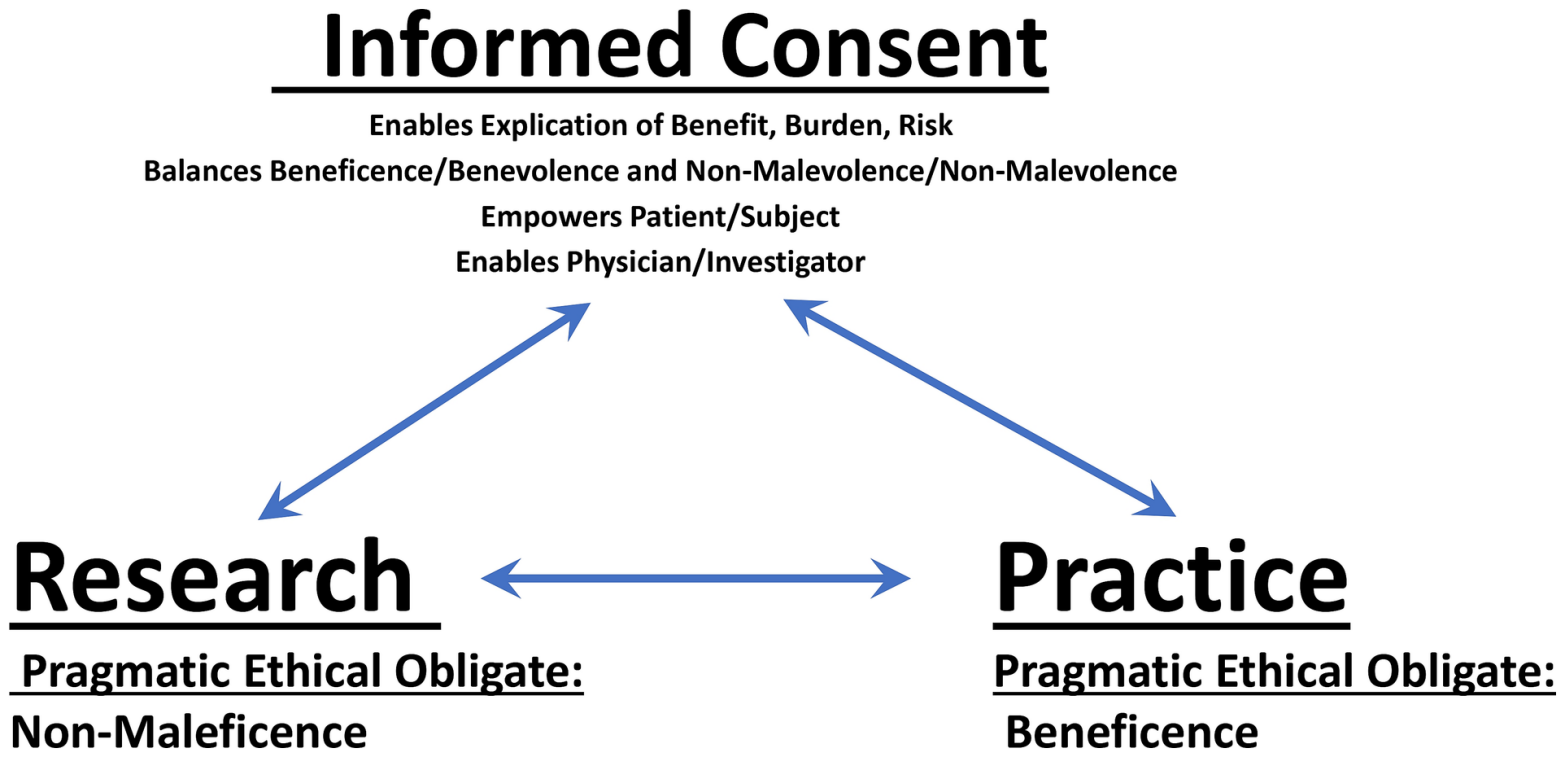
Components and interactions of informed consent in clinical research.

The findings, effects, utility, and value of the neurosciences are undeniably important, consequential and meaningful. Thus, by definition, brain research can be regarded as untrivial. In light of such gravitas, it is critical that the conduct of such research must be ethically sound, so as to sustain its rectitude and probity—both as instrumental to medical practice, and to sustain its worth as a social good. While the primary ethical dictate of clinical medical practice is beneficence (i.e., attaining good); the realistic ethical keel of research is non-maleficence (i.e., preventing or mitigating harm), given that the intended good ends of investigative work are never assured. For research that is performed in clinical practice, ethical probity is afforded by informed consent, which bridges the aforementioned imperatives of benevolence (viz., desiring to attain the good)/beneficence (i.e., achieving good), and non-malevolence (viz., desiring to prevent or mitigate harm)/non-maleficence (i.e., reducing harm)—as focal to therapeutic and investigative endeavors, respectively—and in so doing, empowers the subject/patient and enables the clinician/researcher (Giordano, 2015) (Figure 9).

Fully informed consent (i.e., providing as much available information as is relevant to the best interests of the patient/subject) is especially obligate in those situations wherein research is entailed within clinical therapeutics. In such circumstances, it is important to communicate: (1) that the exploratory activities of research may not inherently benefit the individual patient/subject (but rather may afford altruistic benefit to the collective public health); (2) that the tenuous nature of research may not allow accounting for (unanticipated) burdens, risks or harms that might actually be incurred; and (3) if and to what extent the individual patient/subject will be assessed and clinically cared for should burdensome or adverse effects of research interventions occur (Giordano, 2016). Contingent for truly informed consent is the veritable explanation of current knowns, unknowns, and provision(s) for continuity of evaluation and therapeutic support (Rossi et al., 2014). As well, given the clearly asymmetrical relationship of the physician/researcher and patient/subject, it is recommended that (1) the physician/principal investigating researcher provide any/all information about the biomedical aspects of any/all procedures, methods, benefits, burdens and risks; and (2) project administrative/patient support professionals be tasked with and responsible for obtaining consent (Okun et al., 2024).

## Mood and neuropsychiatric disorders

### Deep brain stimulation for chronic hallucinations in treatment-resistant schizophrenia

**Figure 10:**
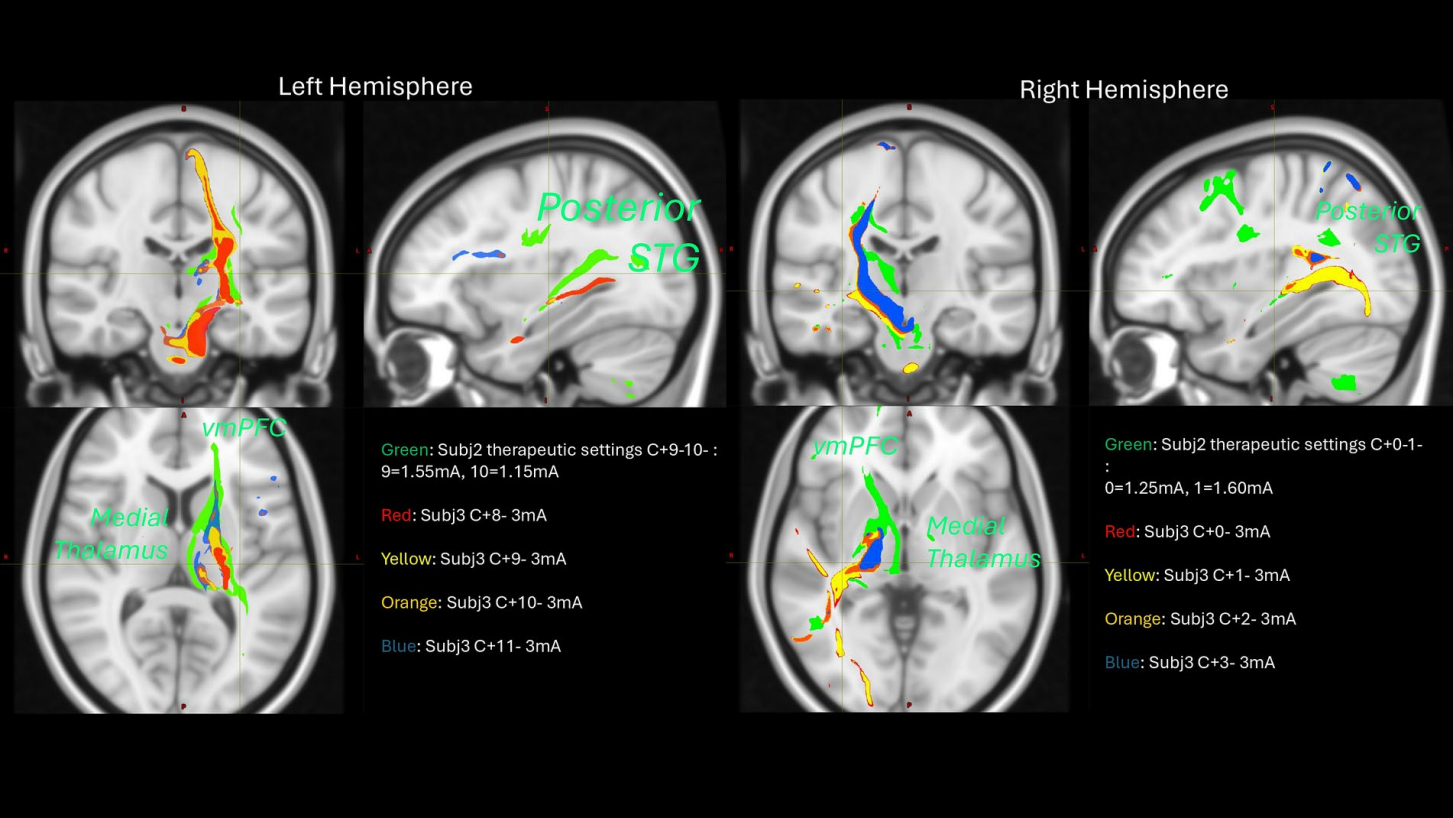
Probabilistic tractography analysis of two subjects across different stimulation configurations.

Among the most characteristic and distressing symptoms of schizophrenia are auditory verbal hallucinations (AVH) that afflict more than 80% of persons with schizophrenia, greatly disturb most patients, and can lead to both suicidal and aggressive behavior. Thus, schizophrenia may be amenable to modulation via DBS. DBS for schizophrenia may hypothetically modulate AVH through its effect on projections from the superior temporal gyrus (STG) to basal ganglia, via the substantia nigra pars reticulata (SNr) and mediodorsal nucleus of the thalamus (MDN). There is ample evidence that this SNr-MDN-STG loop is involved in AVH of schizophrenia. Lesions in this loop can cause new-onset schizophrenia-like AVH. Furthermore, neuroimaging studies suggest that AVH arise from increased activity in speech-sensitive regions of the left posterior STG. Nicola Cascella’s group provides preliminary evidence supporting the hypothesis that bilateral SNr DBS induces acute and permits sustained remission/improvement of otherwise intractable chronic hallucinations in patients with treatment-resistant-schizophrenia. The first patient at the time of initial monopolar programming showed that bilateral stimulation of the SNr produced acute resolution of AVH. After 6 months, her score for hallucinations decreased from baseline 7 (extremely severe) to 1 (no hallucinations reported by the patient) based on the Brief Psychiatric Rating Scale. The patient remains free of hallucinations after 4 years of stimulation onset with a few relapses addressed by changes in stimulations settings. Patient 2 has had a sustained improvement of AVH with impact on her quality of life. Patient 3 had an acute response at the time of the initial programming which was not sustained over time even after several changes in stimulation settings. Further probabilistic tractography analysis showed that patient 3 missed on the left, connections to the MD-STG and ventromedial prefrontal cortex (see Figure 10) that could potentially explain his lack of response. This analysis informed us to program a revised surgery for patient 3 and for the first enrolled patient of the National Institutes for Mental Health sponsored study.

### Capturing mesocorticolimbic electrophysiologic correlates of obsessive-compulsive symptoms to guide a circuit-based deep brain stimulation strategy

DBS for treatment-resistant obsessive-compulsive disorder (trOCD) has shown promise, but its effectiveness can vary. To improve outcomes, Casey Halpern’s group sought to refine the DBS target based on orbitofrontal connectivity given the breadth of data supporting involvement of the orbitofrontal cortex in OCD. As part of their ongoing early feasibility trial of sEEG-guided DBS for trOCD (NCT05623306), they implanted sEEG electrodes in cortical and subcortical targets within the mesocortico-limbic circuit in trOCD including 4 depth electrodes in orbitofrontal cortex. Connectivity is mapped using single-pulse evoked potential. The participants are presented with both provocative images and control images and asked to rate OCD-related distress. Time-frequency analysis of the electrographic data across sEEG electrodes is performed to identify an electrophysiological biomarker of OCD-related distress. The task is then repeated with both sham and circuit-based active stimulation to discern effects of acute DBS. Preliminarily, high-frequency, bipolar stimulation delivered acutely to the ventral basal ganglia appears to robustly modulate orbitofrontal electrophysiology and associated OCD-related distress. These findings suggest that specific circuit-based biomarkers associated with OCD-related provocations can be identified and modulated through DBS. This electrophysiologically-guided strategy may help guide selection of personalized DBS targets in trOCD.

### Does DBS repair brain circuits? Evidence from studies of SCC DBS for TRD

Over the last several years, studies of SCC-DBS for TRD have worked to develop biomarkers that track or predict clinical response (Alagapan et al., 2023; Sendi et al., 2021). Having previously optimized a tractography-guided approach to reliably target and stimulate the confluence of four white matter bundles at the SCC in individual patients (Riva-Posse et al., 2018), these new studies focused on identifying brain changes that explain the observed stereotypical patterns and time course of DBS mediated behavioral effects—namely, an initial improvement in negative mood and psychomotor speed followed by slower progressive changes in overall symptom ratings over weeks to months.

Leveraging the sensing capabilities of prototype DBS systems and machine learning methods, Helen Mayberg’s group developed and validated a SCC LFP biomarker that accurately identifies and tracks the stability of antidepressant response with chronic DBS (Alagapan et al., 2023) (replication unpublished). With the recent safety clearance for multi-sequence MRI scanning with implanted devices, it is now possible to additionally monitor functional and structural network-wide brain changes. This is of particular interest, since a unique feature of SCC DBS for TRD is the stability of clinical effects once achieved with sustained response maintained with ongoing stimulation over many years and slow return of symptoms with treatment discontinuation. Such observations suggest some degree of DBS-induced neuroplastic changes, but direct evidence has been lacking. To test this, resting-state brain scans were acquired using multiple methods (positron emission tomography, functional MRI, diffusion MRI) in 3 patient cohorts at discreet time points over 6 months of treatment. Across methods and cohorts, the function of the default mode network showed the most robust and consistent changes, with the magnitude of increases in cerebral blood flow and glucose metabolism (PET), and normalized Amplitude of Low-Frequency Fluctuations (ALFF, fMRI) significantly correlated with the degree of clinical improvement (Cha et al., 2024). In addition to functional changes, structural changes, indexed by fractional anisotropy (FA), were seen in some but not all stimulated bundles. Supporting these initial findings of selective functional and structural ‘repair’ with chronic SCC DBS, are analogous changes in two male rhesus macaques stimulated unilaterally for 6 weeks at the same confluence of white matter bundles (Fujimoto et al., 2024). These preliminary findings provide new insights into potential network mechanisms mediating long-term sustained effects of SCC DBS and a foundation for similar analyses in other disorders.

## Emerging techniques and DBS

### Parkinson’s DBS targets are part of the somato-cognitive action network

**Figure 11:**
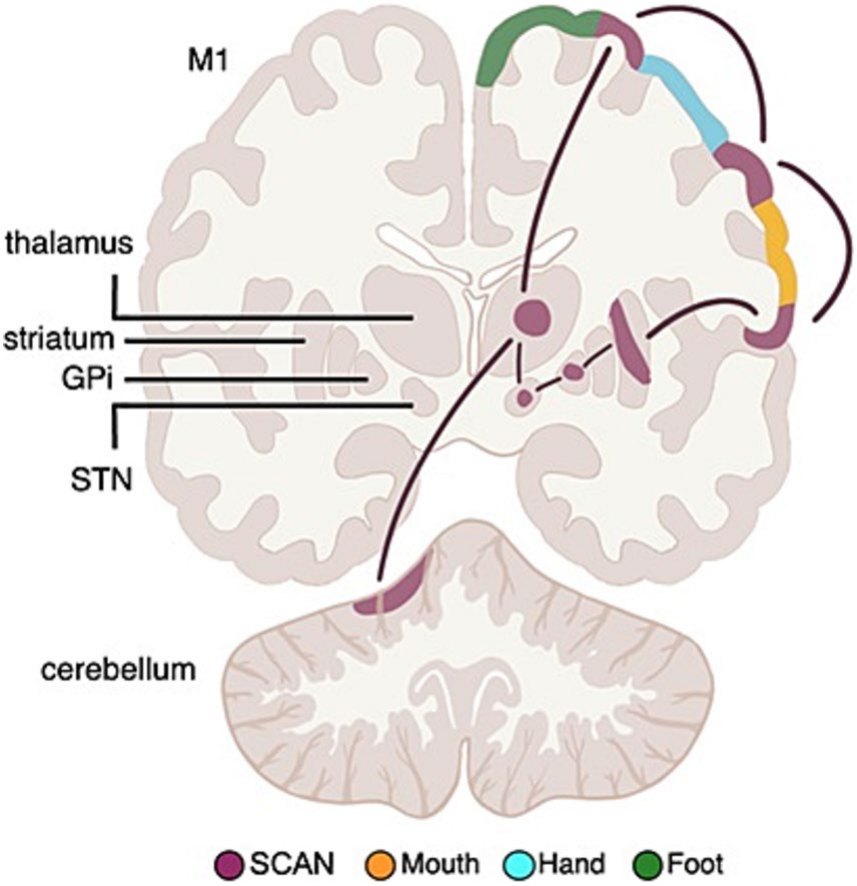
Somato-cognitive action network (SCAN) functional connectivity to subcortex. Resting state functional connectivity (RSFC) analyses in Parkinson’s Disease (PD) patients and healthy controls has revealed that the subcortical circuitry previously mostly associated with classical effector-specific motor functions (foot, hand, mouth), is more strongly connected to the SCAN in cortex. The most important DBS (deep brain stimulation) targets in PD, namely the subthalamic nucleus (STN) and globus pallidus pars interna (GPi) are also part of SCAN, not motor networks.

The newly recognized somato-cognitive action network (SCAN) interleaves with effector-specific primary motor cortex regions (foot, hand, mouth) down the central sulcus (Gordon et al., 2023; Graziano, 2023; Leopold, 2023). The cortical SCAN nodes are strongly functionally connected to each other, and to the action-mode network (AMN) (Dosenbach et al., 2024), critical for action (Dosenbach et al., 2006; Graziano, 2016) and physiological control (Dum et al., 2016; Pool and Ransohoff, 1949), arousal (Dum et al., 2016), errors (Neta et al., 2015) and pain (Hoeppli et al., 2022) (see Figure 11).

PD symptoms cut across motor, physiological and volitional domains [e.g., postural instability, autonomic dysfunction, and reduced self-initiated activity (Bloem et al., 2021; Dauer and Przedborski, 2003)], mirroring SCAN connections to regions relevant for postural control, volition, and physiological regulation (Darby et al., 2018; Pool and Ransohoff, 1949; Siegel et al., 2014; Wall and Davis, 1951). Therefore, Nico Dosenbach’s group investigated whether the PD DBS targets in the STN and GPi are more strongly functionally connected to the SCAN, or to effector-specific motor cortex (Ren et al., 2023).

Resting state functional connectivity (RSFC) data from PD patients and age-matched controls revealed that the STN and GPi are functionally more connected to SCAN than to effector-specific motor cortex, in PD and healthy controls. The selective connectivity to SCAN over effector-specific motor regions held for the STN and GPi (full structure), as well as for their more spatially specific DBS sweet spots (Elias et al., 2021).

The SCAN includes the most important PD DBS targets (Bloem et al., 2021), hence the efficacy of PD-DBS may be tied to modulating functional connectivity between SCAN and subcortical nodes. Thus, Dosenbach proposes that the SCAN should be investigated as the circuit-therapeutic target in PD, not the classical foot, hand, mouth motor networks.

### Delivery of biologic drugs with MR-guided focused ultrasound in Parkinson’s disease

Neuromodulation in PD is traditionally achieved by electrically stimulating or lesioning nodes or connections within brain circuits. Molecular neuromodulation is an alternative approach in which brain circuits are altered by the targeted expression of ion channels, receptors, or neurotransmitters by neurons. Examples include optogenetic techniques, which are commonly used to manipulate brain circuits in experimental models (Scanziani and Häusser, 2009). An example in humans has included glutamic acid decarboxylase expression to enhance GABA-mediated inhibition of the subthalamic nucleus in PD. However, this type of targeted expression to achieve molecular neuromodulation typically requires delivery of viral vectors by direct intraparenchymal injection (LeWitt et al., 2011).

An emerging technology for targeted brain delivery of biologic agents, such as viral vectors, is magnetic resonance-guided focused ultrasound (MRgFUS) at low-intensity in combination with intravenous injection of microbubbles. The microbubbles oscillate when they pass through sonicated tissue, causing mechanical forces that temporarily open the blood–brain barrier (BBB) and thereby facilitate passage of molecules through and into the brain parenchyma. Visualization with MR allows for precise targeting of BBB opening within specific brain regions. This approach has been shown to enhance delivery of trastuzumab, a humanized anti-HER2 receptor monoclonal antibody, to brain metastases in patients with Her2-positive metastatic breast cancer (Meng et al., 2021).

To start investigating the application of this technology to PD, Kalia and colleagues set out to first evaluate the safety and feasibility of using low-intensity MRgFUS with microbubbles to deliver a biologic agent to the unilateral putamen (Huang et al., 2022; Meng et al., 2022). For the biologic agent, they considered various therapies that had previously been demonstrated to be safe with direct intraparenchymal injection, such as growth factor (Whone et al., 2019) or AAV2-neurturin (Marks et al., 2010). They also considered biologics found to be safe when given intravenously but with poor BBB penetration, including anti-*α*-synuclein antibodies (Brys et al., 2019) or recombinant glucocerebrosidase (GCase) (Barton et al., 1991).

Kalia and colleagues selected GCase based upon approximately 30 years of demonstrated safety when delivered intravenously, accessibility of the drug for the study, and biological rationale for its potential to have disease modifying activity in PD. They also selected participants (n = 4) with *GBA1*-related PD since GCase deficiency is a likely pathobiological factor in this genetic subtype of PD. They found that delivery of recombinant GCase to the unilateral putamen using MRgFUS was safe and tolerable in this small first-in-human study. Adverse events were all transient and included headache, symptoms directly related to frame placement, dyskinesia, or T2* signal change on MRI. A phase I/II trial is now underway to investigate bilateral delivery to the putamen in people with *GBA1*-related PD or idiopathic PD (NCT05565443). This research lays the foundation for investigation into this novel modality not just for delivery of disease-modifying therapies in PD, but also for targeted delivery of chemical or biologic agents for molecular neuromodulation.

### Minimally invasive neuromodulation using magnetic nanomaterials

DBS is an effective treatment for neurological and psychiatric disorders. Exploring less invasive alternatives to DBS could broaden its clinical and research applications. Sarah-Anna Hescham’s group investigated minimally invasive, wireless DBS using magnetic nanomaterials that are injectable, battery-less, and completely contained within the brain. Magnetic fields are advantageous for wireless neuromodulation as they can penetrate the skull and brain tissue safely and non-invasively. Achieving cell-specific neuromodulation with these fields requires converting magnetic energy into biologically relevant signals via actuators (Signorelli et al., 2022). Miniaturized actuators, ranging from small isotropic magnetic nanoparticles to larger submicron anisotropic nanomaterials, can produce thermal, mechanical, or electrical stimuli based on the external magnetic field and nanomaterial properties. They demonstrated magnetothermal neuromodulation in freely moving mice by activating the heat-sensitive transient receptor potential cation channel subfamily V member 1 (TRPV1) with synthetic magnetic nanoparticles.

Exposure to an alternating magnetic field causes the nanoparticles to dissipate heat, triggering reversible firing of TRPV1-expressing neurons. This method enabled remote modulation of motor behavior in healthy mice and alleviated motor deficits in parkinsonian models (Hescham et al., 2021). However, magnetothermal DBS faces challenges, including the need for sophisticated power electronics and genetic modification of target neurons. To address these challenges, Hescham’s group also tested magneto-mechanical neuromodulation with magnetite nanodiscs that produce torque under a low frequency alternating magnetic field, activating mechanosensitive ion channel proteins like TRPV4 or PIEZO1.

Hescham’s group observed motor responses from unilateral STN stimulation using magneto-mechanical neuromodulation without genetic modification. Consequently, these techniques enabled remote brain control without the need for hardware or connectors, though their full translational potential remains to be explored.

## Conclusion

The DBS Think Tank XII spotlighted the forefront of neuromodulation technology and its cutting-edge applications. Beyond movement disorders and epilepsy, neuromodulation is now making strides in stroke rehabilitation and psychiatric disorders. Chronic recordings of local field potentials have enabled the decoding of complex sleep patterns, with the potential for optimizing stimulation during sleep. This breakthrough has propelled adaptive DBS technology into its next phase of development and maturation, demonstrating impressive performance when compared to standard clinician programming. MEG has emerged as a powerful tool for studying whole-brain effects of neuromodulation facilitating minimal invasiveness and unparalleled time resolution. This technology has driven the creation of cortical maps using DBS, informing network mapping and has provided an objective assessment of cortical effects.

Industry leaders showcased significant improvements in existing products and introduced upcoming technologies aimed at expanding access and clinical use. The long-term pipeline features innovative approaches such as magnetic nanoparticles and low-intensity MR-guided focused ultrasound. Finally, as advancements accelerate, it is crucial to critically analyze the ethical and social impacts of these therapies. We must consider the systemic consequences of device abandonment and the untriviality of brain biopsies as well as other emerging techniques.

## Data Availability

The original contributions presented in the study are included in the article/Supplementary material, further inquiries can be directed to the corresponding author.

## Supplementary material

SUPPLEMENTARY VIDEO S1 DBS-evoked cortical potentials over time. Grand-average of evoked potentials across 110 recordings with different stimulation settings applied to the left subthalamic nucleus in 22 persons with Parkinson’s disease. The absolute z-scored amplitudes are shown on a cortical MNI template (ICBM125 2009c Nonlinear Asymmetric) using a threshold of 3.0 (z-score) for visualization purposes as indicated by the white line in the color bar. After a baseline period of 10 ms [−10, 0 ms], the DBS pulse creates a high amplitude signal across both hemispheres (time point 0). From about 3–4 ms onwards the cortical distribution of evoked potentials can be observed over time (unpublished data from Bahners et al., 2023).
