# Corrigendum: Proceedings of the 12th annual deep brain stimulation think tank: cutting edge technology meets novel applications

**DOI:** 10.3389/fnhum.2025.1612584

**Published:** 2025-05-06

**Authors:** Alfonso Enrique Martinez-Nunez, Christopher J. Rozell, Simon Little, Huiling Tan, Stephen L. Schmidt, Warren M. Grill, Miroslav Pajic, Dennis A. Turner, Coralie de Hemptinne, Andre Machado, Nicholas D. Schiff, Abbey S. Holt-Becker, Robert S. Raike, Mahsa Malekmohammadi, Yagna J. Pathak, Lyndahl Himes, David Greene, Lothar Krinke, Mattia Arlotti, Lorenzo Rossi, Jacob Robinson, Bahne H. Bahners, Vladimir Litvak, Luka Milosevic, Saadi Ghatan, Frederic L. W. V. J. Schaper, Michael D. Fox, Nicholas M. Gregg, Cynthia Kubu, James J. Jordano, Nicola G. Cascella, YoungHoon Nho, Casey H. Halpern, Helen S. Mayberg, Ki Sueng Choi, Haneul Song, Jungho Cha, Sankaraleengam Alagapan, Nico U. F. Dosenbach, Evan M. Gordon, Jianxun Ren, Hesheng Liu, Lorraine V. Kalia, Sarah-Anna Hescham, Dorian M. Kusyk, Adolfo Ramirez-Zamora, Kelly D. Foote, Michael S. Okun, Joshua K. Wong

**Affiliations:** ^1^Norman Fixel Institute for Neurological Diseases, University of Florida, Gainesville, FL, United States; ^2^School of Electrical and Computer Engineering, Georgia Institute of Technology, Atlanta, GA, United States; ^3^Movement Disorders and Neuromodulation Centre, University of California San Francisco, San Francisco, CA, United States; ^4^Medical Research Council Brain Network Dynamics Unit, Nuffield Department of Clinical Neurosciences, University of Oxford, Oxford, United Kingdom; ^5^Departments of Biomedical Engineering, Electrical and Computer Engineering, Neurobiology and Neurosurgery, Duke University and Duke University Medical Center, Durham, NC, United States; ^6^Department of Neurobiology, Duke University Medical Center, Durham, NC, United States; ^7^Department of Neurosurgery, Duke University Medical Center, Durham, NC, United States; ^8^Center for Neurological Restoration, Cleveland Clinic, Cleveland, OH, United States; ^9^Department of Neurology, Cleveland Clinic Lerner College of Medicine of Case Western Reserve University, Cleveland, OH, United States; ^10^Weill Cornell Medical College, Feil Family Brain and Mind Research Institute, New York, NY, United States; ^11^Restorative Therapies Group Implantables, Research, and Core Technology, Medtronic Inc., Minneapolis, MN, United States; ^12^Department of Neurosurgery, University of California, Los Angeles, CA, United States; ^13^Boston Scientific Neuromodulation, Valencia, CA, United States; ^14^Neuromodulation Division, Abbott, Plano, TX, United States; ^15^NeuroPace, Inc., Mountain View, CA, United States; ^16^Newronika SpA, Milan, Italy; ^17^West Virginia University, Morgantown, WV, United States; ^18^Department of Bioengineering, Rice University, Houston, TX, United States; ^19^Department of Electrical and Computer Engineering, Rice University, Houston, TX, United States; ^20^Department of Neurology, Brigham & Women's Hospital, Harvard Medical School, Center for Brain Circuit Therapeutics, Boston, MA, United States; ^21^Institute of Clinical Neuroscience and Medical Psychology, Medical Faculty and University Hospital Düsseldorf, Heinrich Heine University Düsseldorf, Düsseldorf, Germany; ^22^Department of Neurology, Center for Movement Disorders and Neuromodulation, Medical Faculty and University Hospital Düsseldorf, Heinrich Heine University Düsseldorf, Düsseldorf, Germany; ^23^Wellcome Centre for Human Neuroimaging, UCL Queen Square Institute of Neurology, London, United Kingdom; ^24^Clinical and Computational Neuroscience, Krembil Research Institute, University Health Network, Toronto, ON, Canada; ^25^Faculty of Medicine, Institute for Neuromodulation and Neurotechnology, University Hospital Tübingen (UKT), University Tübingen, Tübingen, Germany; ^26^Department of Neurosurgery, Mount Sinai Medical Center, New York, NY, United States; ^27^Department of Neurosurgery, Maria Fareri Children's Hospital, Westchester Medical Center, Valhalla, NY, United States; ^28^Department of Neurology, Mayo Clinic, Rochester, MN, United States; ^29^Department of Neurology, Georgetown University Medical Center, Washington, DC, United States; ^30^Department of Biochemistry, Georgetown University Medical Center, Washington, DC, United States; ^31^Neuroethics Studies Program, Georgetown University Medical Center, Washington, DC, United States; ^32^Department of Psychiatry, Johns Hopkins University School of Medicine, Baltimore, MD, United States; ^33^Department of Neurosurgery, University of Pennsylvania, Philadelphia, PA, United States; ^34^Department of Surgery, Corporal Michael J. Crescenz Veterans Affairs Medical Center, Philadelphia, PA, United States; ^35^Nash Family Center for Advanced Circuit Therapeutics, Icahn School of Medicine at Mount Sinai, New York, NY, United States; ^36^Department of Radiology and Neurosurgery, Icahn School of Medicine at Mount Sinai, New York, NY, United States; ^37^Department of Neurology and Psychiatry, Icahn School of Medicine at Mount Sinai, New York, NY, United States; ^38^Department of Neurology, Washington University School of Medicine, St. Louis, MO, United States; ^39^Mallinckrodt Institute of Radiology, Washington University School of Medicine, St. Louis, MO, United States; ^40^Department of Psychological & Brain Sciences, Washington University, St. Louis, MO, United States; ^41^Department of Biomedical Engineering, Washington University, St. Louis, MO, United States; ^42^Program in Occupational Therapy, Washington University, St. Louis, MO, United States; ^43^Department of Pediatrics, Washington University School of Medicine, St. Louis, MO, United States; ^44^Department of Radiology, Washington University School of Medicine, St. Louis, MO, United States; ^45^Changping Laboratory, Beijing, China; ^46^Biomedical Pioneering Innovation Center, Peking University, Beijing, China; ^47^Edmond J Safra Program in Parkinson's Disease, Krembil Research Institute, Toronto Western Hospital, University Health Network, Toronto, ON, Canada; ^48^Division of Neurology, Department of Medicine, University of Toronto, Toronto, ON, Canada; ^49^School for Mental Health and Neuroscience, Maastricht University, Maastricht, Netherlands; ^50^Department of Neurosurgery, Maastricht University Medical Center, Maastricht, Netherlands; ^51^Department of Neurosurgery, RWTH Aachen University Hospital, Aachen, Germany

**Keywords:** neuromodulation, deep brain stimulation, Parkinson's disease, epilepsy, sleep, stroke, depression, obsessive-compulsive disorder

In the published article, there was an error in the **author list** and author Sarah-Anna Hescham was erroneously excluded. The corrected author list appears below.

“Alfonso Enrique Martinez-Nunez^1*^, Christopher J. Rozell^2^, Simon Little^3^, Huiling Tan^4^, Stephen L. Schmidt^5^, Warren M. Grill^5,6^, Miroslav Pajic^5^, Dennis A. Turner^5,6,7^, Coralie de Hemptinne^1^, Andre Machado^8,9^, Nicholas D. Schiff^10^, Abbey S. Holt-Becker^11^, Robert S. Raike^11^, Mahsa Malekmohammadi^12,13^, Yagna J. Pathak^14^, Lyndahl Himes^14^, David Greene^15^, Lothar Krinke^16,17^, Mattia Arlotti^16^, Lorenzo Rossi^16^, Jacob Robinson^18,19^, Bahne H. Bahners^20,21,22^, Vladimir Litvak^23^, Luka Milosevic^24,25^, Saadi Ghatan^26,27^, Frederic L. W. V. J. Schaper^20^, Michael D. Fox^20^, Nicholas M. Gregg^28^, Cynthia Kubu^8^, James J. Jordano^29,30,31^, Nicola G. Cascella^32^, YoungHoon Nho^33^, Casey H. Halpern^33,34^, Helen S. Mayberg^35,36,37^, Ki Sueng Choi^35,36^, Haneul Song^35^, Jungho Cha^35^, Sankaraleengam Alagapan^2^, Nico U. F. Dosenbach^38,39,40,41,42,43^, Evan M. Gordon^44^, Jianxun Ren^45^, Hesheng Liu^45,46^, Lorraine V. Kalia^47,48^, Sarah-Anna Hescham^49,50,51^, Dorian M. Kusyk^1^, Adolfo Ramirez-Zamora^1^, Kelly D. Foote^1^, Michael S. Okun^1^ and Joshua K. Wong^1^.”

The authors apologize for this error and state that this does not change the scientific conclusions of the article in any way. The original article has been updated.

